# A rechargeable iodine-carbon battery that exploits ion intercalation and iodine redox chemistry

**DOI:** 10.1038/s41467-017-00649-7

**Published:** 2017-09-13

**Authors:** Ke Lu, Ziyu Hu, Jizhen Ma, Houyi Ma, Liming Dai, Jintao Zhang

**Affiliations:** 1 Key Laboratory for Colloid and Interface Chemistry, Ministry of Education, School of Chemistry and Chemical Engineering, Shandong University, Jinan, 250100 China; 2 College of Science, Beijing University of Chemical Technology (BUCT), Beijing, 100029 China; 3 BUCT-CWRU International Joint Laboratory, College of Energy, Beijing University of Chemical Technology, Beijing, 100029 China; 4 Center of Advanced Science and Engineering for Carbon (Case4carbon), Department of Macromolecular Science and Engineering, Case Western Reserve University, 10900 Euclid Avenue, Cleveland, OH 44106 USA

## Abstract

Graphitic carbons have been used as conductive supports for developing rechargeable batteries. However, the classic ion intercalation in graphitic carbon has yet to be coupled with extrinsic redox reactions to develop rechargeable batteries. Herein, we demonstrate the preparation of a free-standing, flexible nitrogen and phosphorus co-doped hierarchically porous graphitic carbon for iodine loading by pyrolysis of polyaniline coated cellulose wiper. We find that heteroatoms could provide additional defect sites for encapsulating iodine while the porous carbon skeleton facilitates redox reactions of iodine and ion intercalation. The combination of ion intercalation with redox reactions of iodine allows for developing rechargeable iodine–carbon batteries free from the unsafe lithium/sodium metals, and hence eliminates the long-standing safety issue. The unique architecture of the hierarchically porous graphitic carbon with heteroatom doping not only provides suitable spaces for both iodine encapsulation and cation intercalation but also generates efficient electronic and ionic transport pathways, thus leading to enhanced performance.

## Abstract (web summary)

Carbon-based electrodes able to intercalate Li^+^ and Na^+^ ions have been exploited for high performing energy storage devices. Here, the authors combine the ion intercalation properties of porous graphitic carbons with the redox chemistry of iodine to produce iodine–carbon batteries with high reversible capacities.

## Introduction

Over the past decades, Li-ion batteries (LIBs), consisting of a lithium-containing cathode and a graphite anode, have demonstrated great success for energy storage via the classic lithium intercalation process^1–4^. However, the specific capacity of LIBs is limited by the cathode, which is still far below the theoretical capacity of graphite (372 mAh g^−1^) and has to be significantly improved in order to meet the ever increasing energy storage demand^5–8^. Along with intensive research on the exploitation of advanced cathode materials of novel chemical and/or physical structures^9–11^, many other rechargeable batteries, such as Na-ion battery^12, 13^, Li–S battery^4^, and Li-/Na-iodine battery^14–16^ have been devised. Recent publications reported rechargeable Li–iodine batteries with a high theoretical energy density/discharge capacity of 612 Wh kg^−1^/211 mAh g^−1^ based on the highly reversible electrochemical redox reaction between the lithium anode and iodine cathode (2Li + I_2_ ↔ 2LiI)^14, 15^. Although the abundance of iodine resource in ocean^14–18^ makes the rechargeable Li–iodine batteries particularly interesting as low-cost, but efficient, alternatives to LIBs, inexpensive and highly conducting iodine-based cathodes with a stable and high iodine loading still need to be developed. So far, a few carbon hosts, including porous carbons, have been developed for Li–iodine batteries^14, 15, 18, 19^. However, the use of heteroatom-doped/functionalized carbon supports to enhance the iodine loading, and hence the battery performance, has been rarely discussed. As for LIBs, the practical application of Li–iodine batteries is also still hindered by the safety risk intrinsically associated with the metallic lithium electrode due to the lithium dendrite formation and its high activity with moisture^1, 3^.

Carbon-based electrodes have been widely used for energy storage/conversion with their performance being strongly dependent on the composition and microstructure of the carbon materials^19–23^. In this context, heteroatom (e.g., B, N, P, and S) doping of graphitic carbons has been proven to significantly improve the electrocatalytic activities for metal-air batteries and enhance the capacity and cycling stability of Li–S batteries^13, 24–26^. Specifically, three-dimensional (3D) conductive porous carbon networks, when used as the anode, have been demonstrated to improve the energy storage performance of lithium/sodium ion batteries by enhancing the ion intercalation^27^. On the other hand, pre-lithiation/sodiation of a carbon anode provides a lithium/sodium reservoir to tune the cell potential^28, 29^. However, it still remains a challenge to incorporate the ion intercalation with redox reactions, for example associated with iodine, to enhance the performance of rechargeable full batteries. Additionally, kinetic balance between anode and cathode still remains elusive. Therefore, it is highly desirable to rationally design electrode materials intercalated with redox active moieties^28–32^ to overcome the discrepancy between cathodic and anodic kinetics, in particular, and to enhance the battery performance, in general.

Herein, we report a free-standing hierarchically porous carbon matrix co-doped with nitrogen and phosphorus (HPCM-NP) prepared by pyrolysis of polyaniline coated cellulose wiper in the presence of phytic acid as the phosphorous source. The resultant free-standing conductive HPCM-NP renders the facile preparation of iodine-containing cathodes (i.e., iodine-carbon cathode) free from current collector, conductive additive, or any additional binder. The highly porous structure, coupled with the heteroatom co-doping, ensures a remarkably high iodine loading up to 125 wt%. Our results reveal that both the chemical and physical structures of HPCM-NP play important roles in regulating the iodine adsorption and subsequent electrochemical performance. Rechargeable Li–iodine and Na–iodine batteries based on the iodine-containing HPCM-NP cathodes exhibit a high discharge capacity of 386 and 253 mAh g^−1^, respectively, along with a good rate-performance and good cycle stability with 84.5% capacity retention after 2000 cycles for the Li–iodine battery and 85.0% capacity retention after 500 cycles for the Na–iodine battery. To avoid the use of the unsafe metallic Li and Na anode, we further constructs rechargeable batteries from the iodine-carbon cathode (I_2_-HPCM-NP) and carbon cloth anode (HPCM-NP) with an electrolyte containing lithium (sodium) ions (e.g., LiTFSI, NaClO_4_), which exhibits a reversible capacity of 217 (182) mAh g^−1^, high energy density of 166 (153) Wh kg^−1^ and good cycle performance with a 76.7% capacity retention after 500 cycles (69.8%@300 cycles) at a current density of 500 mA g^-1^. Our kinetics and mechanistic studies reveal that proper encapsulation of iodine into the HPCM-NP electrodes with a hierarchically porous structure and a controllable heteroatom doping level could efficiently enhance the Li-/Na-ion intercalation and electrolyte diffusion whilst restoring a kinetic balance between the anode and cathode electrodes, leading to the good battery performance.

## Results

### Structure characterization of iodine-carbon composite

**Fig. 1:**
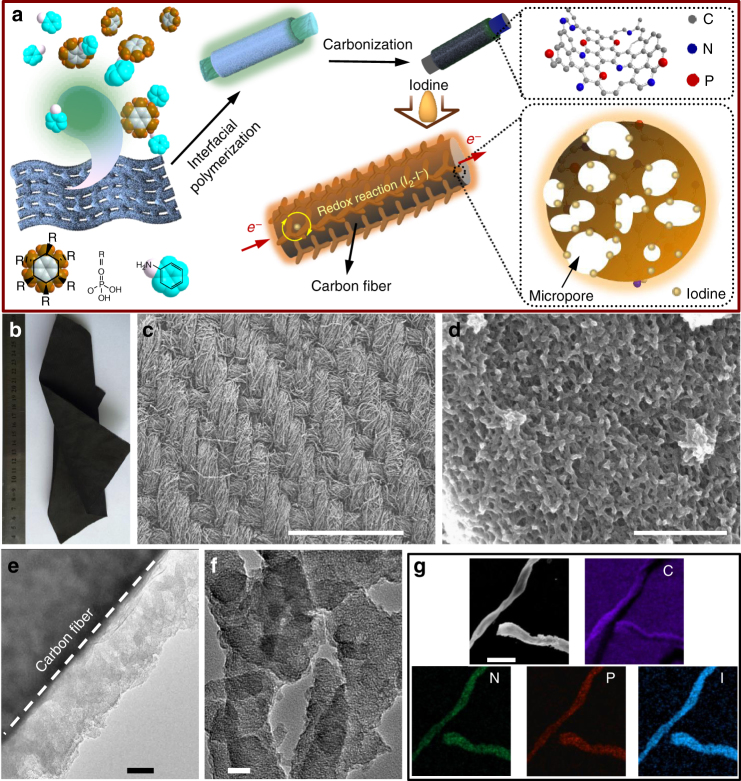
Preparation of HPMC-NP and the loading iodine. **a** Interfacial deposition of PANi on the cellulose wiper via an oxidative polymerization in the presence of phytic acid and the subsequent carbonization in N_2_ atmosphere. Then, iodine is escasulated into the as-prepared HPMC-NP from the iodine-saturated solution. **b** The digital photograph, **c**, **d** SEM images (*Scale Bar*, 1 mm and 500 nm, respectively), and **e**, **f** TEM images of the free-standing HPCM-NP (*Scale Bar*, 100 nm and 20 nm, respectively). **g** SEM image (*Scale Bar*, 15 μm) and the corresponding elemental mapping of the iodine loaded HPCM-NP

**Fig. 2:**
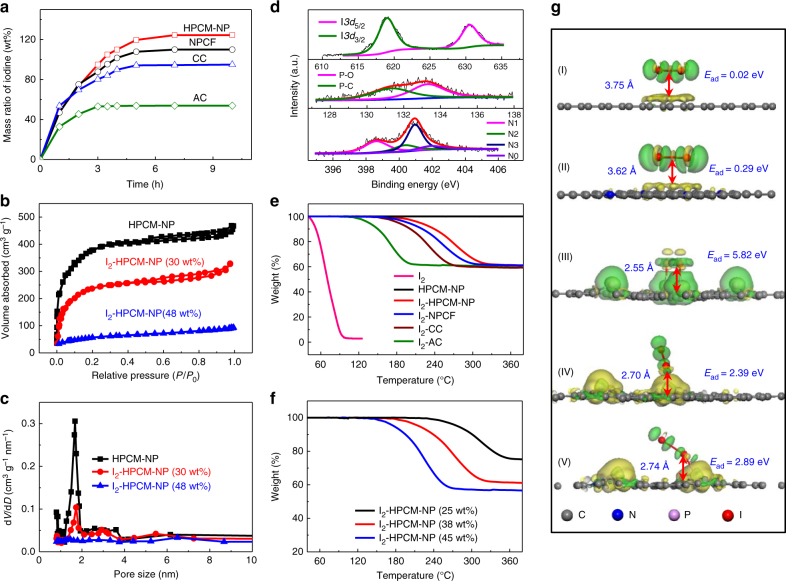
Compositional characterization and stability analysis of iodine–carbon composites. **a** The time-dependent profiles for the mass ratio of iodine adsorbed on various carbons (the mass ratio is normalized to the mass of carbon). **b** N_2_ adsorption-desorption isotherms and **c** the corresponding pore size distribution curves of HPMC-NP with different mass loading of iodine. **d** High-resolution XPS spectra of N1*s*, P2*p*, and I3*d* for I_2_-HPCM-NP samples. Thermogravimetric analysis curves of **e** pure iodine and iodine-carbon composites (iodine content, 40 wt%), and **f** I_2_-HPCM-NP composite with different iodine content. **g** The contour plots of the difference in charge density for the optimized structures of iodine molecule adsorbed on graphene I, graphene doped with N (II), P (III), isolated N and P (IV), and coupled N and P (V). The differential charge density was calculated from: Δ*ρ* = *ρ*_12_−*ρ*_1_−*ρ*_2_, where *ρ*_1_, *ρ*_12_ and *ρ*_2_ are the chare density of iodine, doped graphene with and without iodine adsorbed on the surface, respectively. *Yellow* and *green color* indicate the charge depletion and accumulation, respectively. The adsorption energies for I_2_ molecule on these fragments were obtained using: *E*_ad_ = −(*E*_1_ + *E*_2_−*E*_12_), where *E*_ad_ is the adsorption energy of the I_2_ molecule on the corresponding surfaces, *E*_1_ is the total energy of the graphene (or heteroatom doped graphene), *E*_2_ is the energy of one isolated I_2_ molecule, and *E*_12_ is the energy of the optimized structures for I_2_ molecules adsorbed on the graphene planes. The bond lengths and heights of adsorbed I_2_ molecule are also listed in the figure, along with the corresponding adsorption energies

Figure 1a schematically illustrates the preparation process for HPCM-NP through an interfacial polymerization of aniline on a cellulose wiper in the presence of phytic acid, followed by carbonization. The oxidative polymerization of aniline monomers in the presence of phytic acid led to the formation of porous polyaniline (PANi) along individual fibers in the cellulose wiper. The subsequent pyrolysis of PANi coated cellulose wiper at an elevated temperature resulted in the formation of HPCM-NP. The optimized condition for the formation of HPCM-NP was determined by adjusing the amount of aniline monomers (Supplementary Figs. 1, 2 and Supplementary Note 1). Then, iodine can be facily loaded into the porous carbon matrix via surface adsorption from an iodine saturated aqueous solution (see Methods for detailed procedure). Large-scale of free-standing and highly flexible HPCM-NP scaffold can be prepared as its size is mainly determined by the size of the starting cellulose wiper (Fig. 1b). Scanning electron microscopy (SEM) and transmission electron microscopy (TEM) images show that the flexible HPCM-NP knits with flexible ligaments (Fig. 1c), and the individual carbon fibers are coverved uniformly with the hierarchical porous carbon network (Figs. 1d, e). The high-resolution TEM image further reveals that the shells of these interconnected fibers contain a myriad of micropores (Fig. 1f, *cf*. Figs. 2b, c). For comparison, carbonized pure cellulose wiper and PANi aerogel were also prepared at the same elevated temperature (Supplementary Figs. 3, 4). To load iodine, these as-prepared carbon scaffolds were immersed into the iodine saturated aqueous solution at room temperature (Supplementary Fig. 5). The hierarchically porous structure with a large surface area facilitated the penetration of the iodine solution into the inside of carbon matrix, possibly by an energtic capilary action^15, 19, 33^, and the adsorption of iodine species throughtout the entire carbon matrix homogenously rather than accumulate on the outmost surface only^4, 34^. This was confirmed by the element mapping analysis along the whole carbon fiber (Fig. 1g) and the EDX spectrum for the bulk sample (Supplementary Fig. 6). The same procedure was used to load iodine into other carbon materials, including the pure porous carbon cloth (CC) (Supplementary Fig. 5), N and P co-doped porous carbon foam (NPCF) (Supplementary Fig. 4) and activated carbon (AC) (Supplementary Fig. 7 and Supplementary Note 2). It was noticed that the co-doping of HPMC-NP with N and P could enhance the uploading of iodine species (*cf*. Fig. 2a)^14, 34^. The hierarchically porous frameworks of HPMC-NP can act as effective physical barriers for preventing the dissolution of the adsorbed iodine (Supplementary Fig. 8), but efficient pathways for electron transfer via the highly conductive 3D carbon skeleton^34, 35^, enhancing the electrochemical performance.

As shown in Fig. 2a, the adsorption of iodine on all the carbon materials investigated in this study followed the same trend: initially increased with increasing time and then leveled off at the equilibrium point. The time to reach the adsorption equilibrium was around 7, 7, 5, and 3 h for HPCM-NP, NPCF, CC, and AC, respectively, and the adsorption capacity at equilibrium decreased from 125 to 50 wt% along the same order (The mass ratio of iodine is normalized to carbon). For AC, the equilibrium was reached rapidly due to the surface-dominated adsorption. In contrast, the adsorption process of iodine on hierarchically porous HPCM-NP is relatively slow^15, 35^, but achieves a large adsorption capacity. X-ray diffraction (XRD) pattern reveals the presence of broad peaks at around 24.5° and 43.6° attributable to the (002) and (101) diffraction peaks of graphitic cabons (Supplementary Fig. 9a). However, no diffraction peak of the loaded iodine is observed, suggesting the formation of non-crystalline iodine^15, 34, 35^. The pronounced Raman D and G bands (~1355 and 1596 cm^−1^, Supplementary Fig. 9b) are ascribed to the disordered carbon and graphitic *sp* ^2^ carbon, respectively. The *I*_D_/*I*_G_ Raman peak intensity ratios were found to be 1.5, 1.4, 1.3, 1.2, and 1.0 for the I_2_-HPCM-NP, I_2_-NPCF, iodine-nitrogen doped porous carbon cloth (I_2_-NCC, see Methods), I_2_-CC, and I_2_-AC, respectively. Thus, it was evident that the formation of highly porous structure and the introduction of heteroatom dopings led to an increased number of edges and surface defects^13^, which most likely improved the iodine adsorption and battery performance^13, 25, 26^. In consistence with the XRD data, Raman spectroscopy reveals no iodine peak, indicating, once again, the formation of amorphous iodine^34, 36^.

Nitrogen adsorption-desorption isotherms show a sharp adsorption slope at *P/P*_*0*_  <  0.1, suggesting the presence of micropores (Fig. 2b and Supplementary Fig. 10). The corresponding pore size distribution curve for HPCM-NP further confirms the presence of micropores with a narrow size distribution (centered at ~1.7 nm, Fig. 2c), attractive for iodine adsorption^33, 35^. Nevertheless, the uptake of iodine led to a significantly decreased specific surface area (i.e., 973 m^2^ g^−1^, vs. 1487 m^2^ g^−1^ for pure HPCM-NP). The same tendency was also observed for other carbon materials (1102, 478, 855, and 205 m^2^ g^−1^ for the NPCF, I_2_-NPCF, CC, and I_2_-CC, respectively). More importantly, the pore size gradually decreased with increasing amount of the adsorbed iodine (Fig. 2c). These results indicate the adsorption/infiltration/accumulation of iodine into the pores^34, 36^. With the iodine loading increased up to ~48 wt%, the specific surface area of HPCM-NP decreased from 1487 to 85 m^2^ g^−1^ (Fig. 2b) with an containment decrease in the volume fraction of micropores (Fig. 2c).

X-ray photoelectron spectroscopy (XPS) survey spectra reveal the presence of C, N, P (Supplementary Fig. 11a), confirming the formation of nitrogen and phosphorus co-doped carbon for HPCM-NP. As expected, the presence of some oxygen (5.1 %) cannot be ruled out^25^. High-resolution N1*s* XPS spectrum of HPCM-NP (Fig. 2d) can be convoluted into four peaks centered at ∼398.6, 400.5, 401.3 and 402.0 eV corresponding to pyridinic (N1), pyrrolic (N2), graphitic (N3), and oxidized pyridinic (N0) nitrogen^13, 25, 37^, respectively. The fitted P2*p* peaks of HPCM-NP located at 131.8 and 133.4 eV can be assigned to the P-C and P-O bonds^25, 26^. Upon the iodine uploading, three peaks centered at 48.5, 619.2, and 630.6 eV were observed (Supplementary Fig. 11b), attributable to I4*d*, I3*d*_5/2_ and I3*d*_3/2_ of iodine (Fig. 2d)^15^. The obvious binding energy shift in comparison with the pure iodine suggests the strong interaction of iodine with the carbon substrate (Supplementary Fig. 12 and Supplementary Note 3). According to the theoretical results (Supplementary Fig. 13), the charge modulation due to the nitrogen and phosphorus doping would contribute to the binding shift, which could also enhance the stability of iodine adsorbed^15, 25, 38^.

The thermal gravimetric analysis (TGA) profile of pure iodine shows the evaporation of iodine blow 80 °C due to its low sublimation temperature (Fig. 2e). Compared with pure iodine, iodine-adsorbed HPCM-NP (I_2_-HPCM-NP) sample shows an increased temperature range (120–200 °C) for iodine evaporation, indicating the strong interaction of iodine with the porous N, P co-doped carbon scaffold^34, 39^. I_2_-HPCM-NP exhibited the highest on-set evaporation temperature at around 200 °C, suggesting the best thermal stability of adsorbed iodine on the HPCM-NP, followed by I_2_-NPCF ( ~ 180 °C), I_2_-CC (~ 150 °C), and I_2_-AC (~ 120 °C). The enhanced thermal stability for the uploaded iodine could lead to a better cycling stability of relevant batteries^15^. For I_2_-HPCM-NP, the on-set temperature for iodine evaporation significantly decreased from ~ 250 to 150 °C with increasing iodine loading (Fig. 2f). Our theoretical calculation reveals that the strong interaction between iodine and graphene can be achieved via N and P doping (Fig. 2g, explained in detail in Supplementary Fig. 13 and Supplementary Note 4). Specifically, the synergic effect of N and P co-doping significantly improves the adsorption energy (*E*_ad_), which will enhance the stability of iodine adsorbed. Furthermore, the strong interactions toward iodine would also benefit to the nucleation of iodine by lowering the surface tensile against the carbon substrate, leading to the high iodine loading^24, 40–42^. With increasing iodine loading, however, more and more iodine molecules aggregated in the pores without directly supported by the carbon surface, resulting in the poorer thermal stability (Supplementary Fig. 14). Therefore, hierarchically porous structure and heteroatom doping are crucial for the stability of adsorbed iodine, which would contribute to the battery performance and it’s cycling stability.

### Electrochemical performance of lithium-iodine battery

**Fig. 3:**
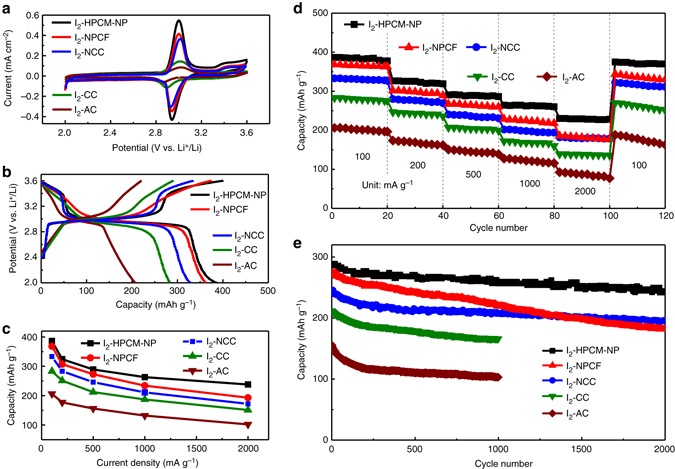
Electrochemical performance of different iodine cathodes for Li–I_2_ batteries. **a** Cyclic voltammograms (0.1 mV s^−1^), **b** charge/discharge voltage profiles (100 mA g^−1^), and **c** specific discharge capacities of different iodine-carbon cathodes between 2.0 and 3.6 V with the same iodine loading of 2.0 mg cm^−2^. **d** Rate capability and **e** cycling stability tests of iodine-carbon cathodes at a current density of 500 mA g^−1^

Cyclic voltammogram (CV) curves show a support-dependent property for various iodine loaded carbon electrodes (Fig. 3a). The well-defined redox peaks in each CV curve indicate a two-step redox process of iodine-carbon composite electrodes. The sharp peak at about 3.0 V and a hump at around 3.4 V correspond to the oxidation of LiI to LiI_3_ and then to higher-order elemental iodine, respectively. In contrast, the cathodic peaks centered at ~ 2.9 and 3.3 V arise from the reversible conversion pairs of LiI_3_/LiI and I_2_/LiI_3_^14, 15^. The differential capacity plot (Supplementary Fig. 15) also confirms the two-step process for the redox reactions. The good linear relationship between the peak current (*i*_p_) and the square root of scan rates for LiI_3_/LiI redox pair (Supplementary Fig. 16) suggests a diffusion-controlled redox process^14^. In contrast, the oxidation current of I_3_^−^ converted to elemental iodine is directly proportional to the value of the scan rates, and thus the ion diffusion is quick and its redox kinetic is only limited by electron transfer. Obviously, the pseudocapacitive character is involved in the redox process of iodine, which would be favorable for both high capacity and high-rate performance^14, 15, 18^.

Notably, the I_2_-HPCM-NP electrode shows the largest current density with good reversibility and the peak potential difference (Δ*E*_p_) of the redox peaks is only 88 mV (Fig. 3a). In contrast, the Δ*E*_p_ of I_2_-NPCF, I_2_-NCC, I_2_-CC, and I_2_-AC is around 91, 135, 170, and 220 mV, respectively. The observed remarkably large current density and good reversibility for the I_2_-HPCM-NP could be attributated, once again, to the synergistic effect of N, P co-doping to introduce more edge/active defects with respect to other carbon hosts^25, 43, 44^. As revealed by the experimental and theoretial results (Supplementary Figs. 13, 17, 18 and Supplementary Note 5), the strong anchoring effect could not only accelerate the nucleation of iodine and enable fast kinetics according to the basical growth behavior of nanostructures, but also stablize iodine and LiI_3_ to retard the shuttle effect, thus improving the reversibility and cycling stability^14, 15, 34^. Therefore, a rational design and controllable construction of the carbon electrode is essential to enhance the overall performance of the electrode materials^13, 34^.

The typical galvanostatic charge/discharge curves (Fig. 3b) of the Li–iodine battery using different iodine-carbon cathodes exhibit two voltage slops at 3.3–3.5, 2.9–3.1 V for charging curves and 3.2–3.4, 2.8–3.0 V for discharging curves. The good symmetric charge/discharge profile for I_2_-HPCM-NP electrode with high Coulombic efficiency (CE) suggests a good reversibility. At the current density of 100 mA g^−1^, the I_2_-HPCM-NP electrode delivered the largest initial discharge capacity of 386 mAh g^−1^ and charge capacity of 391 mAh g^−1^ with a high CE of 98.7% and high symmetry (Fig. 3b and Supplementary Fig. 19). Such a high capacity is even larger than the theoretical capacity (211 mAh g^−1^) for Li–iodine batteries^14, 15^. This is because the pure HPCM-NP would also contribute to the whole capacity of I_2_-HPCM-NP due to the capacitive contribution (~56 mAh g^−1^ in Supplementary Fig. 20, the details in Supplementary Note 6)^14, 15^. Notably, the specific capacity of I_2_-HPCM-NP decreased with increasing the iodine loading (Supplementary Fig. 21) since not all iodine participated in the redox reactions at high loadings. At the mass loding of 2 mg cm^−2^, the calculated discharge capacities for I_2_-HPCM-NP, I_2_-NPCF, I_2_-NCC, I_2_-CC and I_2_-AC at different current densities from 100 to 2000 mA g^−1^ are summarized in Fig. 3c.

The initial specific capacities for I_2_-NPCF, I_2_-NCC, I_2_-CC, and I_2_-AC cathodes are around 368, 331, 283, and 206 mAh g^−1^, respectively, at a current density of 100 mA g^-1^. These capacities decreased to 238, 179, 151 and 102 mAh g^−1^ when the current density increased to 2000 mA g^−1^, corresponding to a capacity fade of 35.3, 45.9, 46.6, and 50.5%. Notably, a good capacity retention of 70.7% was observed for I_2_-HPCM-NP after a total of 100 cycles at the increased current density from 100, 200, 500, 1000 to 2000 mA g^−1^ (20 consective cycles at each of the current densities; Fig. 3d). Thereafter, the capacity can be recovered to 375 mAh g^−1^ with a capacity retention of 97.2% by switching the current denisty back to 100 mA g^−1^, which is better than the corresponding values for other composite electrodes (96.7, 93.5, 95.4, and 91.3% capacity retention for I_2_-NCC, I_2_-NPCF, I_2_-CC, and I_2_-AC, respectively). The 3D hierarchical porous framework not only allows for the electrolyte to easily access into the inner surface but also ensures effective electronic transport along the conductive carbon skeleton (Supplementary Fig. 22 and Supplementary Note 7). Thus, the improved rate capability for the I_2_-HPCM-NP can be achieved. Besides, I_2_-HPCM-NP samples obtained at various temperatures 600-1000 °C exhibited increased specific capacity from 215 to 295 mAh g^−1^ with increasing temperature (Supplementary Fig. 23), possibly due to the formation of graphitic carbon with a good electric conductivity at high pyrolysis temperatures^13, 25^. A long-term cycling stability test for I_2_-HPCM-NP revealed a capacity retention of 84.5% after 2000 charge/discharge cycles (79.4 and 66.3% capacity retention after 2000 cycles for I_2_-NCC and I_2_-NPCF; 78.3 and 66.0% capacity retention after 1000 cycles for I_2_-CC and I_2_-AC), suggesting the good cycling stability (Fig. 3e). The cycling performance of I_2_-HPCM-NP is thus comparable to many other similar iodine cathodes in the literature (Supplementary Table 1)^14, 15, 18, 19^. It is revealed a slow dissolution of iodine into the electrolyte (Supplementary Fig. 24 and Supplementary Note 8) during the cycling test and no obvious side reaction of the electrolyte is observed except the revisible redox of iodine over the potential range tested (Supplementary Fig. 25 and Supplementary Note 9). However, the gradual formation of Li dendritics on the Li-metal electrode (Supplementary Fig. 26 and Supplementary Note 10) could slightly deteriorate the battery stablity. In addition to the strong anchoring effect of heteroatom doping, the improved cycling stability should be attributed to the rational-designed porous carbon matrix prepared via the direct pyrolysis of 3D PANi aerogel on a carbon fiber substrate without nonconductive binders, which provides abundant electrode/electrolyte contact interfaces and reduces ion diffusion path for fast electrochemical kinetics (Supplementary Fig. 22)^13, 24, 25, 34^. In constrast, for those reported binder-added electrodes, additional irreversible capacity loss and poor cycling stability are inevitable, arising from the insulating, inactive, and easily swelling polymeric binders^45^.

### Electrochemical performance of sodium-iodine battery

**Fig. 4:**
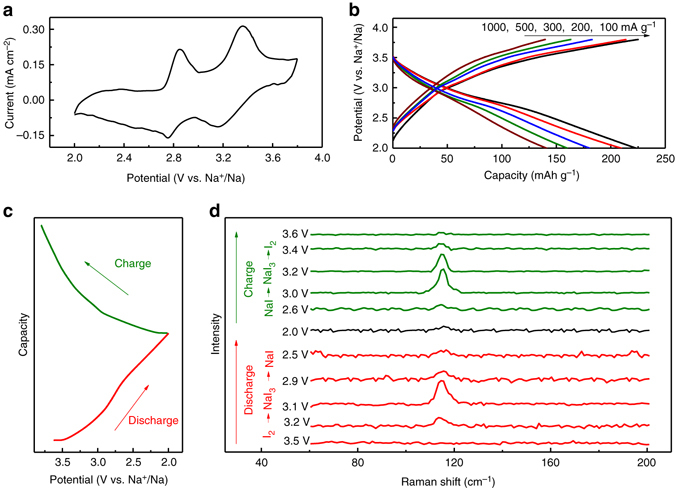
Electrochemical performance of I_2_-HPCM-NP cathode for a Na–I_2_ battery. **a** CV curve (0.1 mV s^−1^) and **b** representative charge/discharge curves of I_2_-HPCM-NP cathode for Na–I_2_ battery. **c** Discharge and charge curves with the test positions for analysis. **d** In situ Raman analyses of Na–I_2_ batteries at different discharge and charge stages

To exploit the broad applications for the iodine-carbon cathodes, I_2_-HPCM-NP electrode was also coupled with Na metal to fabricate a Na–I_2_ battery. The CV curve (Fig. 4a) exhibits a two-step redox process. Typically, the two cathodic peaks located at 3.15 and 2.76 V could be ascribed to the sequential reduction transitions of I_2_/I_3_^−^ and I_3_^−^/I^−^ with the insertion of Na^+^ ions. In the reverse process, the two anodic peaks are ascribed to the oxidation of NaI to NaI_3_ at 2.75 V and the subsequent oxidation to I_2_ at 3.16 V^16^. The typical charge/discharge curves (Fig. 4b) reveal that the specific capacity is 224, 200, 171, 156 and 137 mAh g^−1^ at a current density of 100, 200, 300, 500 and 1000 mA g^−1^, respectively. The specific capacity is smaller than that of Li–I_2_ battery at the same current density (vide supra) due to the larger ion radius and slower diffusion kinetics for Na^+^ ions^12, 13, 16, 28^. To gain deep insight into the energy storage process in a Na–I_2_ battery, we performed in-situ Raman measurements at various discharging/charging stages (Figs. 4c, d). During the discharge process, a characteristic peak at about 115 cm^−1^ emerged and gradually became more intense as the reaction progressed. This peak could be ascribed to the symmetric stretching mode of I_3_^−^, corresponding to the conversion of I_2_ to I_3_^−^ along with the insertion of Na^+^ ions^15, 16^. With further decreasing potential to 2.0 V, the gradually disappeared peak indicated the reaction transformation of NaI_3_ to NaI at the second reaction step. Therefore, it was evident that the reversible reactions occurred during the discharging/charging processes via 2Na + I_2_ ↔ 2NaI with the formation of NaI_3_ as an intermediate^14–16^. These results indicate that the redox behaviors of iodine and sodium couple is quite similar to those of Li–I_2_ battery. When the current density was increased by ten times (100 to 1000 mA g^−1^), a capacity retention of ~61.2% was obtained and ~93.8% of the initial discharge capacity (210 mAh g^−1^) can be recovered when the current was restored to 100 mA g^−1^ (Supplementary Fig. 27a), suggesting a good rate-performance. Besides, the Na–I_2_ battery retained 85% of its initial discharge capacity over 500 charge/discharge cycles (Supplementary Fig. 27b), exhibiting also a good cycling stability^14–16, 19^.

### Electrochemical performance of iodine–carbon full batteries

As demonstrated above, the specific capacity of Li–I_2_ battery is larger than that of Na–I_2_ battery, though the same I_2_-carbon cathode was used. Therefore, the ion intercalation at the carbon electrode should have a significant influence on its specific capacity, presumably due to the different sizes between Li and Na ions^15, 16^. When used as anodes in LIBs, the first discharging capacities are around 388, 375, 369, 211, and 163 mAh g^−1^ for HPCM-NP, NPCF, NCC, CC, and AC (Supplementary Fig. 28), respectively. The observed good performance of the HPCM-NP anode (Supplementary Fig. 29 and Supplementary Note 11) is comparable to and even better than those of other carbon-based anodes (see Supplementary Table 2), particularly coupled with LiMn_2_O_4_ and Na_3_V_2_(PO_4_)_3_/C cathodes (Supplementary Fig. 30 and Supplementary Note 12) in full batteries using Li^+^/Na^+^ ion containing electrolytes (Supplementary Fig. 31). These results indicate that HPCM-NP can be also used as anodes in Li/Na-ion batteries based on ion intercalation process, and, if coupled with the I_2_-HPCM-NP cathode, a metal-electrode-free iodine-carbon full battery can be developed by using Li^+^/Na^+^-containing electrolytes.

**Fig. 5:**
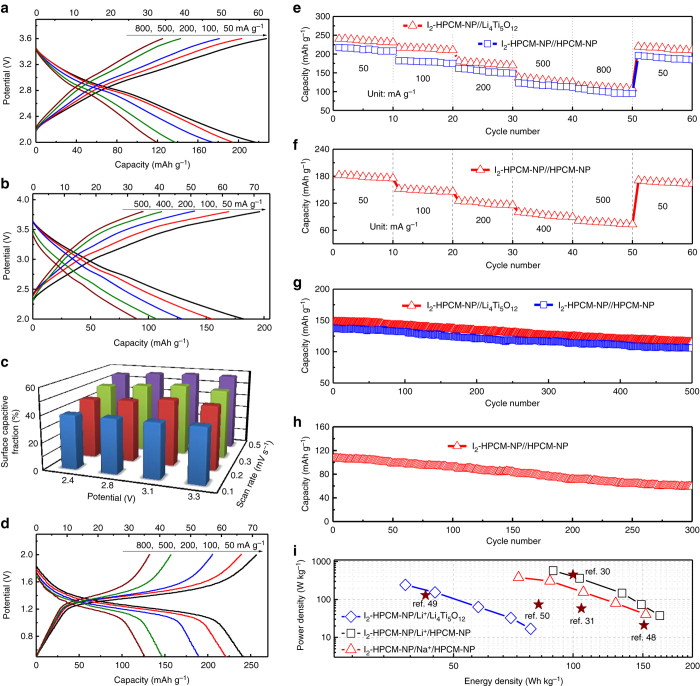
Electrochemical performance of iodine–carbon hybrid full battery. Charge/discharge curves at different rates of I_2_-HPCM-NP//HPCM-NP in **a** LiTFSI and **b** NaClO_4_, respectively, and the corresponding discharge capacities (*up*: based on the weight of anode and cathode; *down*: based on the weight of carbon-based electrodes). **c** Surface pseudocapacitance (redox) contributions at different conditions. **d** Charge/discharge curves at different rates of I_2_-HPCM-NP//Li_4_Ti_5_O_12_ full battery and the corresponding discharge capacities (*up*: based on the weight of anode and cathode; *down*: based on the weight of carbon-based electrodes). **e**, **f** Rate capability and **g**, **h** cycling performance of different hybrid full batteries with **e**, **g** LiTFSI and **f**, **h** NaClO_4_. **i** Ragone plot of full batteries, where power and energy densities are estimated based on the total mass of cathode and anode materials

To further incorporate the redox reactions of iodine with the intercalation process, a full battery is fabricated by coupling I_2_-HPCM-NP cathode with HPCM-NP (I_2_-HPCM-NP//HPCM-NP) anode in a Li- or Na-ion electrolyte, which is free from the Li or Na metal anode, and hence without the associated safety risk^7^. The charge/discharge curves of I_2_-HPCM-NP//HPCM-NP exhibited relatively smooth profiles without obvious discharge plateaus for both the Li (LiTFSI, Fig. 5a)- and Na (NaClO_4_, Fig. 5b)- ion electrolyte. The discharge capacity at the current density of 50 mA g^−1^ is 217, 182 mAh g^−1^ in a Li-, Na-ion electrolyte, respectively. The typical CV curves with a couple of redox peaks (Supplementary Fig. 32) show the redox behaviour of iodine coupled with insertion/extraction of Li^+^ ions. To clarify the contributions of both processes, the fractions of redox capacitive and diffusion-controlled contributions to charge storage were determined (Fig. 5c)^46, 47^. At a low scan rate of 0.1 mV s^−1^, the pseudocapacitive contribution was only 40%, indicating that  ~ 60% of the total stored charge was on the basis of the ion intercalation process at a specific potential (e.g., 2.8 V). However, the ratio increased to  ~ 57% at a high scan rate of 0.5 mV s^−1^. These results indicate the higher pseudocapacitive contribution renders the better high-rate performance as the ion intercalation process needs a longer time to achieve^46, 47^. The almost equal contributions ( ~ 50%) for the both processes seen in Fig. 5c suggests that the energy storage of a metal-electrode-free iodine-carbon full battery can be modulated by adjusting both redox reactions of iodine and cation intercalation - a plausible concept, but one which has not yet been realized. To further verify the feasibility, HPMC-NP was replaced with a typical zero-strain intercalative anode, Li_4_Ti_5_O_12_ (Supplementary Fig. 33), in the full cell (Fig. 5d). The CV curve of I_2_-HPCM-NP exhibited a pair of redox peaks of iodine located at around of 3.0 V whereas the peaks for Li_4_Ti_5_O_12_ located at around 1.5 V was ascribed to the reversible intercalation process of Li^+^ ions (Supplementary Fig. 34). When coupled both electrodes in a full cell, a pair of redox peaks at around 1.42/1.27 V could be ascribed to the overall electrochemical reaction (2Li_4_Ti_5_O_12_ + 2xLiI ↔ 2Li_4+x_Ti_5_O_12_ + I_2_), in which both redox reactions and the ion intercalation were coupled together, leading to a specific capacity of 240 mAh g^−1^ (Fig. 5d). The ratio of pseudocapacitive contribution over intercalative one at a given voltage is quantitatively determined according to the CV curves at various scan rates (Supplementary Fig. 35). The calculated capacity contribution from the surface pseudocapacitive is in the range of 41-64%. This result implies a dominating diffusion-limited ion-intercalative process at slow scan rates whereas the surface redox reaction is restricted at a specific potential. Further quantitative capacitive analyses on the hybrid ion storage behavior indicate that the surface pseudocapacitive contribution increased from 41% to 61% with increasing the scan rate at a fixed potential (1.45 V) as the high scan rate could facilitate the pseudocapacitive process (*cf*. Fig. 5c). These results support the concept to improve the performance of I_2_-HPCM-NP//HPCM-NP full batteries by the combination of iodine ion intercalation with its redox reactions.

According to the rate capability tests (Figs. 5e, f), the capacity retentions of I_2_-HPCM-NP//HPCM-NP and I_2_-HPCM-NP//Li_4_Ti_5_O_12_ after 50 cycles at various current densities from 50 to 800 mA g^−1^ (about 16 times increase) were about 49.9 % (108.3 mA h g^−1^) and 48.0% (115.1 mAh g^−1^), respectively (Fig. 5e). For the hybrid full battery in a Na-ion electrolyte, the capacity retentions of I_2_-HPCM-NP//HPCM-NP was approximately 44.6 % (81.1 mAh g^−1^) after 50 cycles with the current densities increased by 10 times (increased from 50 to 500 mA g^−1^) (Fig. 5f). Furthermore, the hybrid full batteries exhibited good cycling stability with capacity retention of ~74% for I_2_-HPCM-NP//HPCM-NP and 76% for I_2_-HPCM-NP//Li_4_Ti_5_O_12_ after 500 cycles at a current density of 500 mA g^−1^ (Fig. 5g). In contrast, the battery in a Na-ion electrolyte can deliver discharge capacity of 58.9 mAh g^−1^ with the initial capacity retention of 55.0% after 300 cycles at a current density of 400 mA g^−1^ (Fig. 5h). The good battery performance in Li-ion electrolyte could be contributed to the smaller size and the fast diffusion kinetics of Li ions^13, 16, 36^. As can be seen from the Ragone plot (Fig. 5i), the power and energy densities of the metal-electrode-free iodine-carbon full batteries are comparable to and even better than recently reported Li-ion or Na-ion full batteries^30, 31, 48–50^. Specifically, energy densities of 165.6 Wh kg^−1^ for the iodine-carbon battery using a Li-ion electrolyte and 152.6 Wh kg^−1^ for that using a Na-ion electrolyte were achieved at the power densities of 37.4 and 40.4 W kg^−1^, respectively. When the electrolyte was included in the calculation, the energy density of full battery was around 27.8/27.0 Wh kg^−1^ in a Li-/Na-ion electrolyte at a power density of 6.5/7.3 W kg^−1^, respectively (Supplementary Fig. 36) because the weight ratio between the electrolyte and electrode materials was in the range of 4.5 ~ 5.0. As shown in Supplementary Fig. 37, a red LED was powered on by an iodine-carbon full battery, which demonstrates that the combination of redox reactions and ion intercalation as an effective approach for the development of high-performance iodine-carbon rechargeable batteries from low-cost heteroatom-doped 3D porous carbon electrodes for efficient energy storage.

## Discussion

In this study, we have prepared 3D free-standing porous carbon matrix co-doped with N and P by a facile pyrolysis of polyaniline coated cellulose wipers generated from interfacial polymerization of aniline on the cellulose wiper in the presence of phytic acid. Owing to the heteroatom-doped hierarchically porous carbon structure, iodine can be efficiently loaded up to a high content (125 wt%), leading to the formation of free-standing iodine-carbon electrodes for fabricating rechargeable Li- and Na–I_2_ batteries with high performance. Typically, the rechargeable Li–I_2_ and Na–I_2_ batteries manifested high discharge capacities of 386 and 253 mAh g^−1^, excellent rate-performances and excellent cycle stabilities (84.5%, 2000 cycles; 85.0%, 500 cycles). More importantly, the combination of the surface-dominated redox reactions of iodine with the intrinsic intercalative properties of such a porous graphitic carbon in a full metallic Li/Na-electrode-free batteries led to high reversible capacities of up to 217/182 mAh g^−1^ for iodine-carbon full batteries with a Li-/Na-ion electrolyte, respectively, and good cycling stabilities (76.7%@500 cycles and 69.8%@300 cycles at a current density of 500 mA g^-1^). The methodology developed in this study opens new avenues for the development of novel rechargeable batteries, even free from the metallic Li/Na anode and associated safety risk, from low-cost heteroatom-doped porous graphitic carbon via the combination of redox capacitive properties and ion intercalation.

## Methods

### Preparation of heteroatom doped porous carbon matrix

1.2 ml aniline monomer was added into 30 ml phytic acid solution. 1.0 g of ammonium persulfate (APS) was dissolved into the 15 mL deionized (DI) water under stirring. After cooling down to about 4 °C, both solutions were mixed together. Cellulose wiper was immersed into above solution and kept at 4 °C for overnight. The resultant wiper was washed with a large amount of DI water and dried at 60 °C, followed by annealing at 1000 °C for 2 h in N_2_. In order to prepare nitrogen doped carbon cloth (NCC), the as-prepared wiper was washed with 10% ammonia water (de-doping process) to remove phytic acid before thermal treatment. For comparison, polyaniline aerogel were also prepared by the same procedure and carbonized at 1000 °C for 2 h in N_2_. Pure carbon cloth was obtained by directly pyrolysis of cellulose cloth under same experimental condition.

### The loading of iodine on various carbon scaffolds

Iodine was loaded on various carbon scaffolds according to a simple inside encapsulation method. Typically, 10 mg of iodine was added into 20 ml DI water. Notably, most of iodine particles remain undissolved at the bottom due to the low solubility. HPMC-NP and other carbon materials were immersed into the above solution for a certain time, respectively. During the inside encapsulation process, it can be seen that most of iodine particles disappeared gradually. Then, the obtained sample was dried at 80 °C. The iodine adsorbed on carbon scaffolds was measured according to the mass change before and after the encapsulation process. The mass loading of iodine was normalized to the total mass of iodine and carbon unless otherwise stated. Notably, the mass ratio in Fig. 2a was based on the mass of carbon.

### Structural characterization

The phase purity and crystal structure of the products were determined by XRD measurements. XPS was recorded on an ESCALAB 250 X-ray photoelectron spectrometer. The Raman spectra were collected on LabRAM HR 800 system using 514 nm laser. SEM images were measured on a Hitachi × 650 electron microscope. TEM images were presented on a JEOL JEM-2100 microscope. The thermostability of iodine-carbon composite was determined by Thermogravimetric analysis (TGA). The heating rate is 5 °C min^−1^ from room-temperature to 400 °C under N_2_. Brunauere-Emmete-Teller (BET) isotherms and specific surface area (i.e., BET surface area) were measure with a Kubo × 1000 instrument at 77 K. Prior to subsequent measurements, the I_2_-loaded porous materials were firstly dried at 80 °C for 10 h to remove the surface adsorbed iodine and water. Then, the samples were degassed at 95 °C for 6 h. The specific surface areas were calculated by the BET method. Pore size distribution curves were computed from the desorption branches of the isotherms using the Barrett, Joyner, and Halenda (BJH) method.

### Electrochemical measurements

The Li–/Na–iodine batteries (half-cell) were assembled in an argon-filled glove box. 1 M LiTFSI in DOL/DME (1:1 by volume) with 1 wt% LiNO_3_ and 1 M NaClO_4_ in EC/DEC (1:1 by volume) were used as the electrolytes, respectively, for Li- and Na–iodine battery test. Cyclic voltammetry (CV) was performed on a CHI 760e electrochemical workstation. The charge and discharge curves were recorded on a Land CT2001A battery test system. Prior to full-battery fabrication, the anode and cathode were electrochemically activated in a half cell, this step is critical to circumvent the large irreversible capacity and the low Coulombic efficiency (CE).

### Computational methods

Density functional theory (DFT) and molecular dynamics (MD) calculations were performed using the Vienna ab initio simulation package^51^. The core-valence interaction was described by the projector augmented-wave (PAW) method^52^. The generalized gradient approximation of Perdew-Burke-Ernzerhof (GGA-PBE)^53^ was used to account for the exchange-correlation functional. The plane-wave energy cutoff was set to 400 eV. The Monkhorst-Pack scheme^54^ was used to sample the Brillouin zone (BZ). For the single layer, a grid of 21 × 21 × 1 was used. The van der Waals (vdW) corrections were used in the calculations of the iodine molecules (I_2_/LiI_3_) adsorption for the systems of pure graphene and graphene doped with N and P. A vacuum region of 20 Å was employed for all the systems to avoid interaction of periodic images. The force convergence criterion was set to 0.01 eV Å^−1^ for optimization.

### Data availability

The relevant data are available within the article and its Supplementary Information file or from the corresponding authors on reasonable request.

## Electronic supplementary material

Supplementary notes
