# Supplementary material for: A rechargeable iodine-carbon battery that exploits ion intercalation and iodine redox chemistry
Source: Nat Commun. 2017 Sep 13;8:527. doi: 10.1038/s41467-017-00649-7 (PMC5597605; doi:10.1038/s41467-017-00649-7)
Supplement: Supplementary file 1 — Supplementary notes [file 41467_2017_649_MOESM1_ESM.pdf]

Description of Supplementary Files

File Name: Supplementary Information
Description: Supplementary Figures, Supplementary Tables, Supplementary Notes and Supplementary References

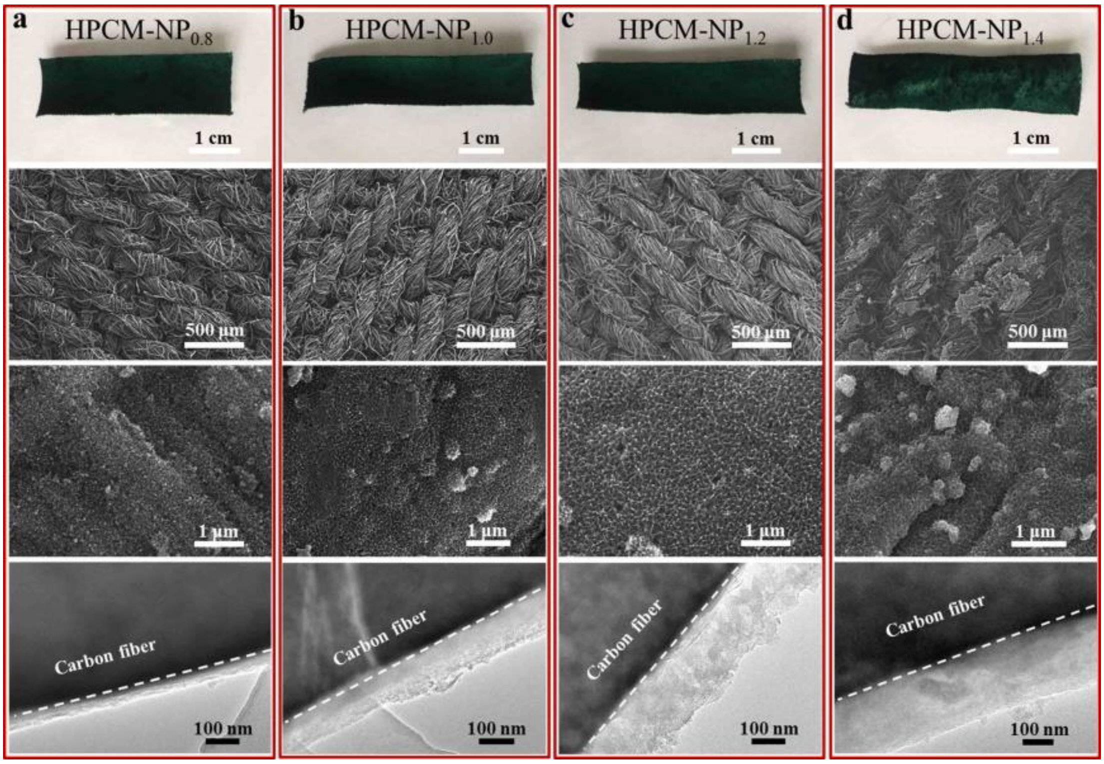

Supplementary Figure 1. Digital photographs and the corresponding SEM and TEM images of HPCM-NP samples prepared with various amounts of aniline monomers (a, 0.8 mL; b, 1.0 mL; c, 1.2 mL; d, 1.4 mL).

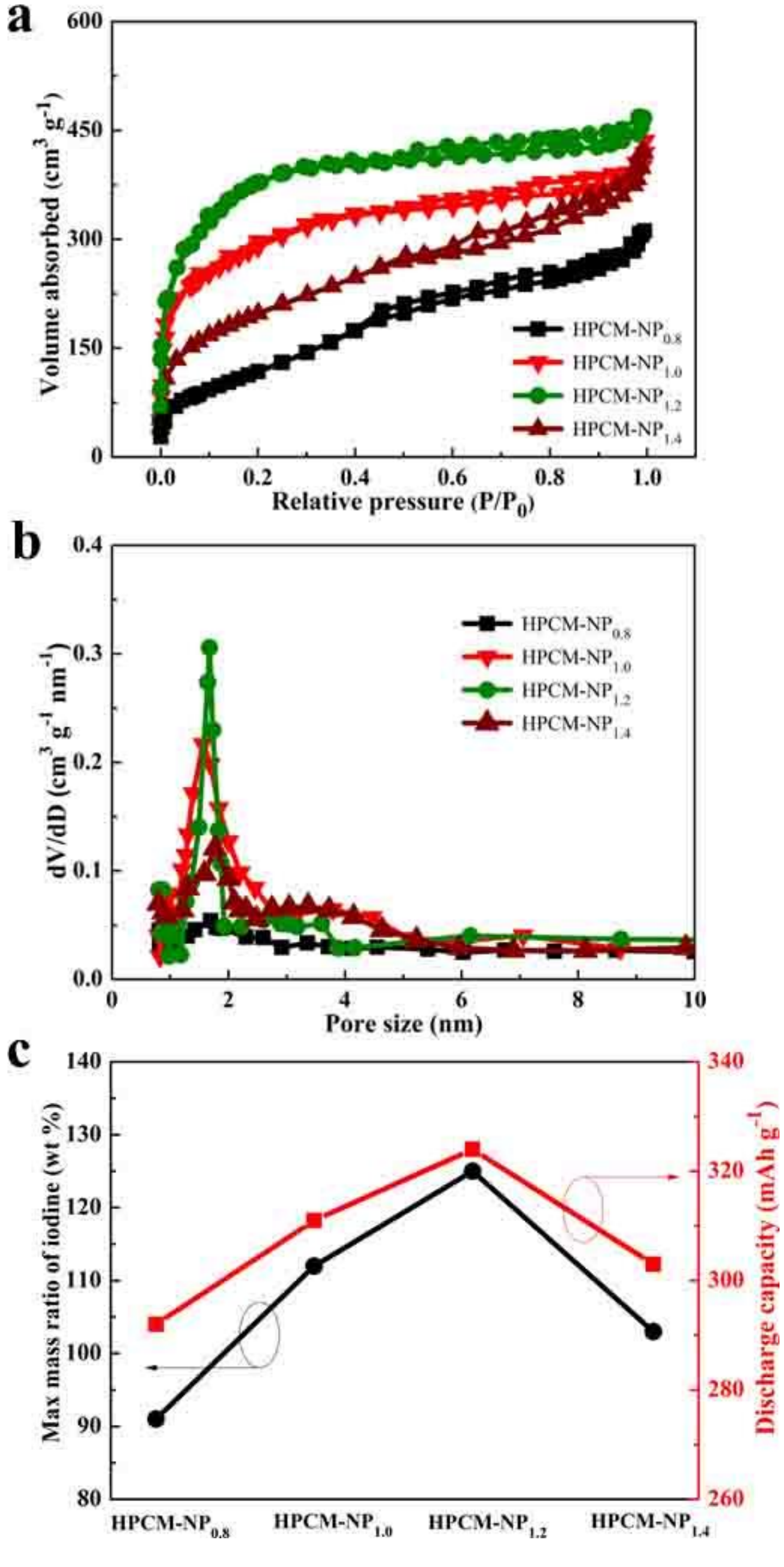

Supplementary Figure 2. (a) N_2 adsorption-desorption isotherms and (b) the pore distribution curves of different HPCM-NP samples. (c) Iodine uptake and specific discharge capacity curves (iodine loading of 2.0 mg cm^{-2}, 200 mA g^{-1}) of different HPCM-NP samples.

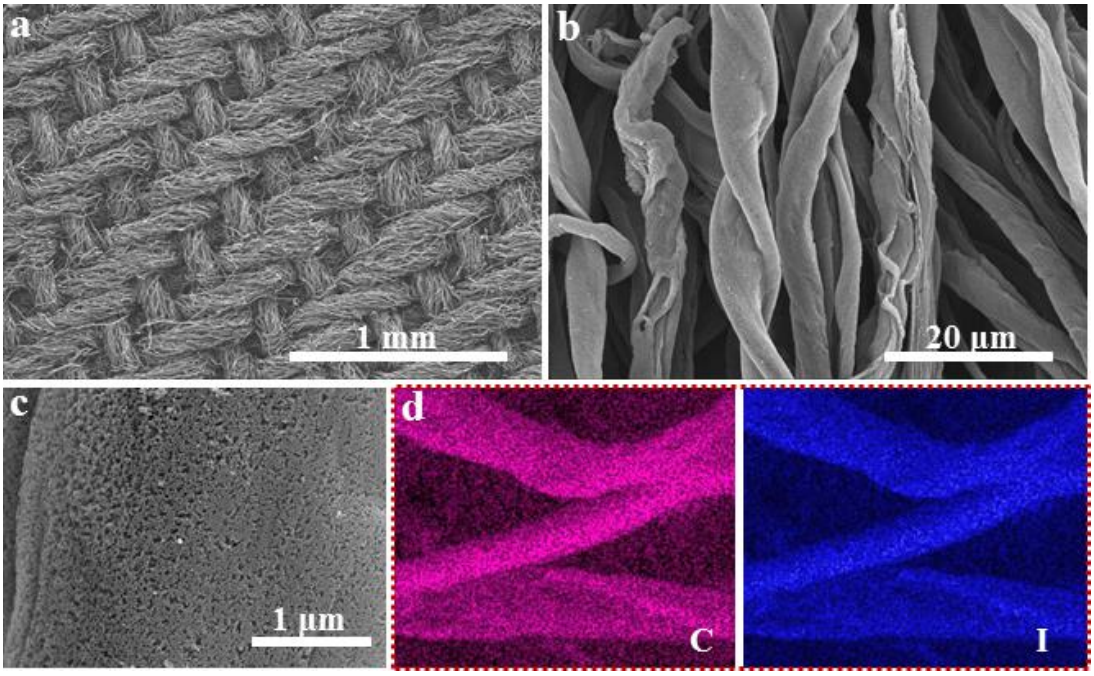

Supplementary Figure 3. (a, b, c) SEM images and (d) elemental mapping of I_2-CC.

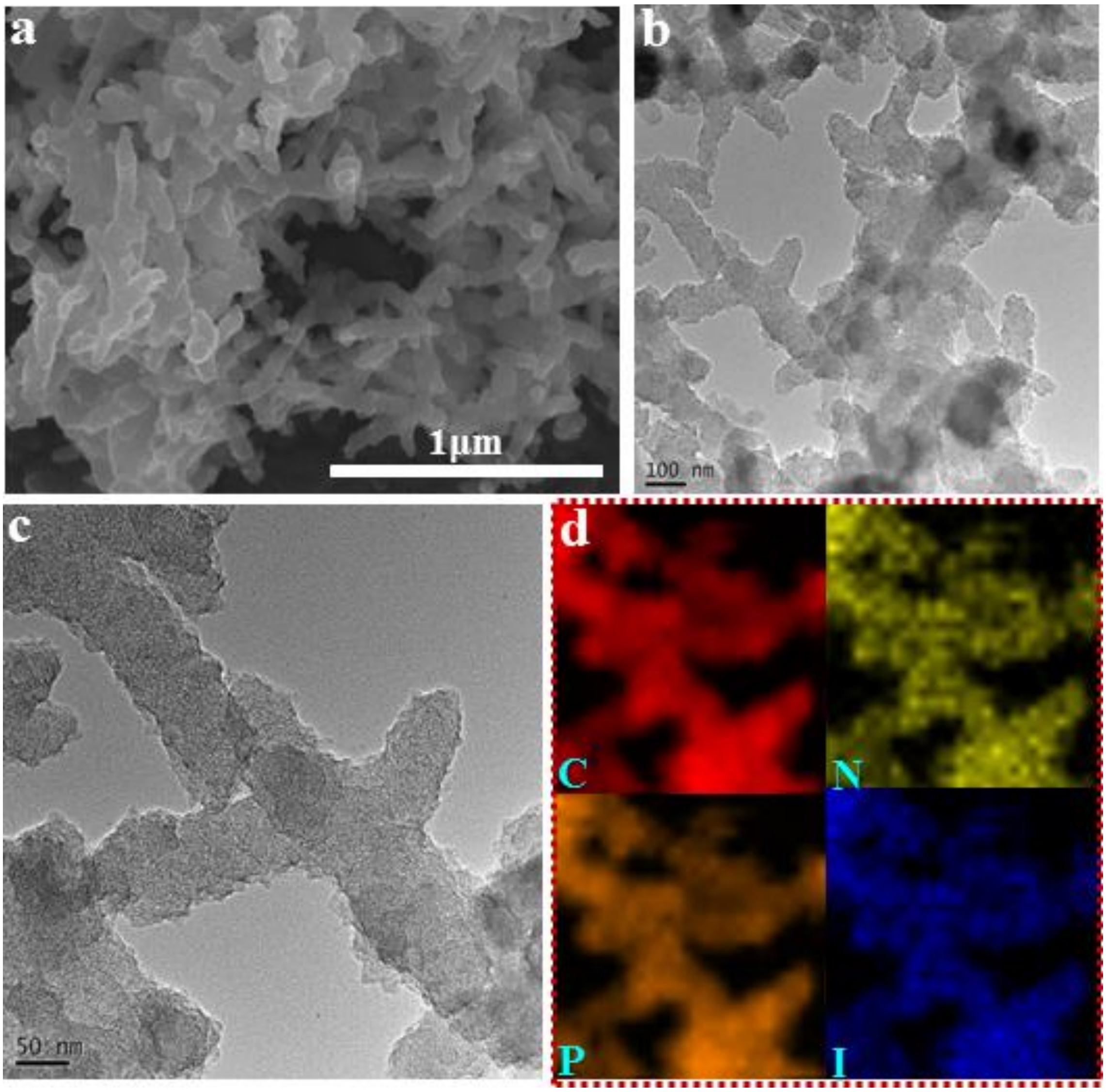

Supplementary Figure 4. (a, b, c) SEM images and (d) elemental mapping of I_2-NPCF.

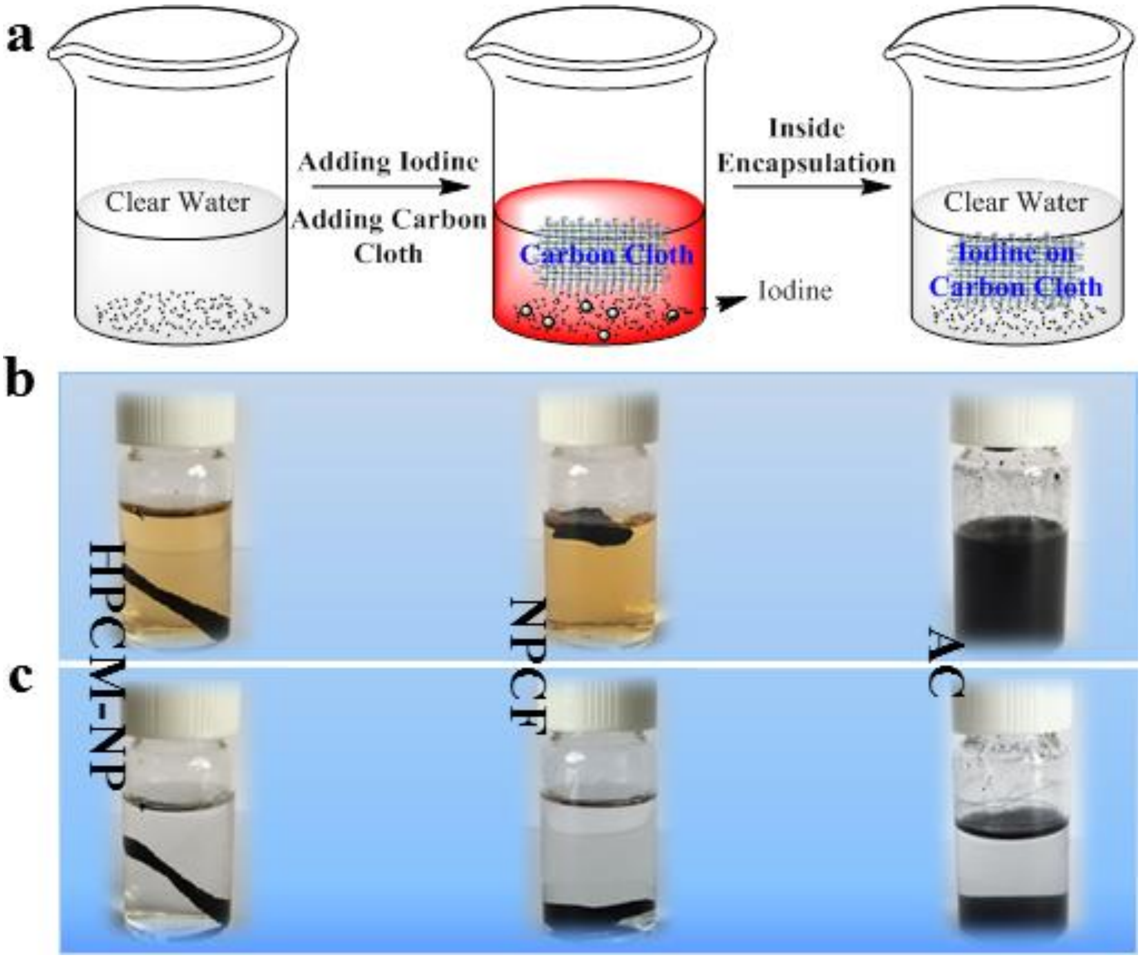

Supplementary Figure 5. (a) Schematic illustration of inside encapsulation process for loading iodine on carbon materials. Digital photo images of carbon materials (b) before and (c) after inside encapsulation process.

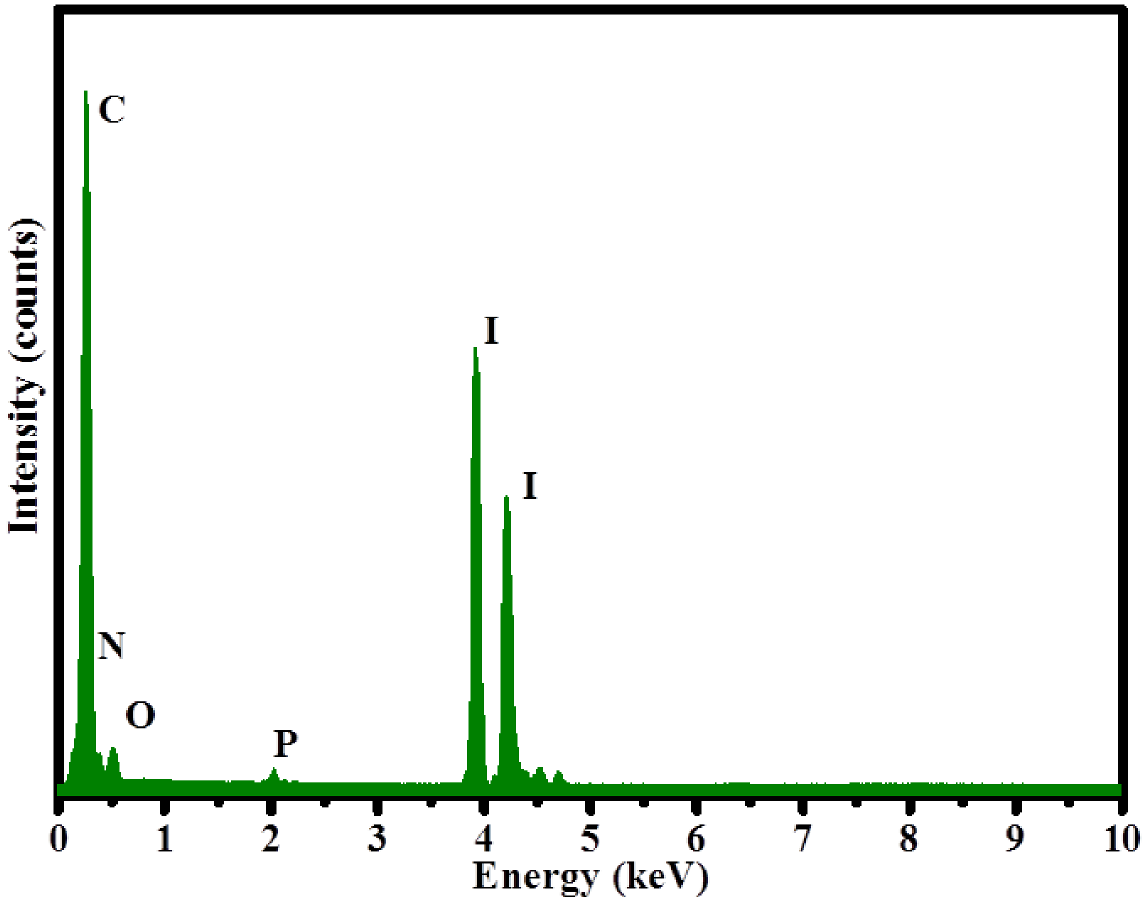

Supplementary Figure 6. Energy-dispersive X-ray (EDX) spectrum of I_2-HPCM-NP.

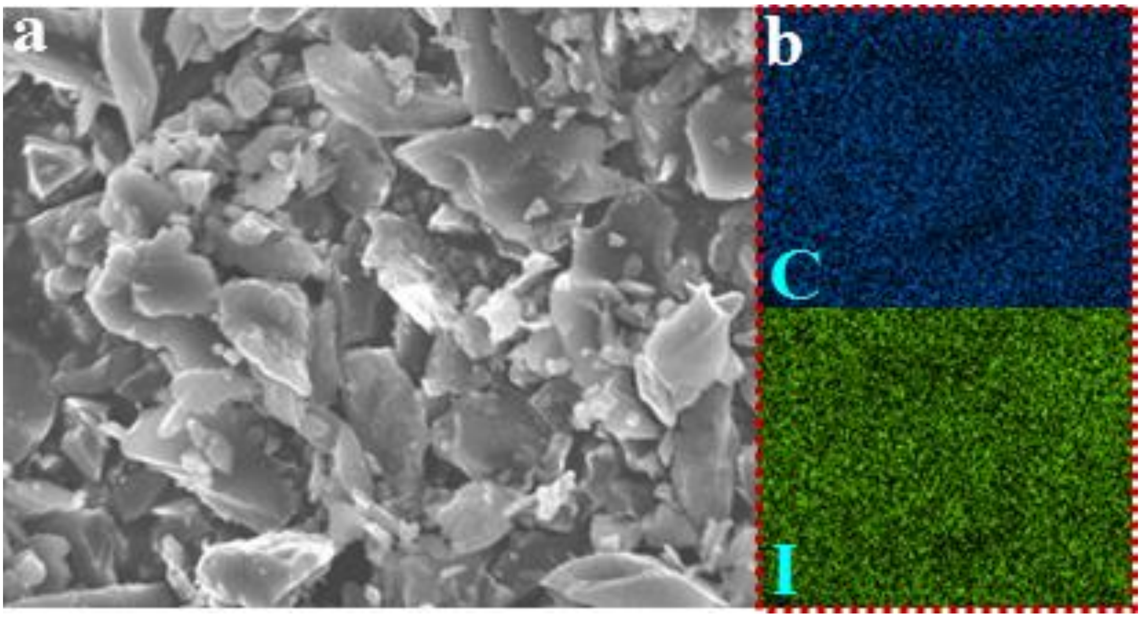

Supplementary Figure 7. (a) SEM image and (b) elemental mapping of I_2-AC.

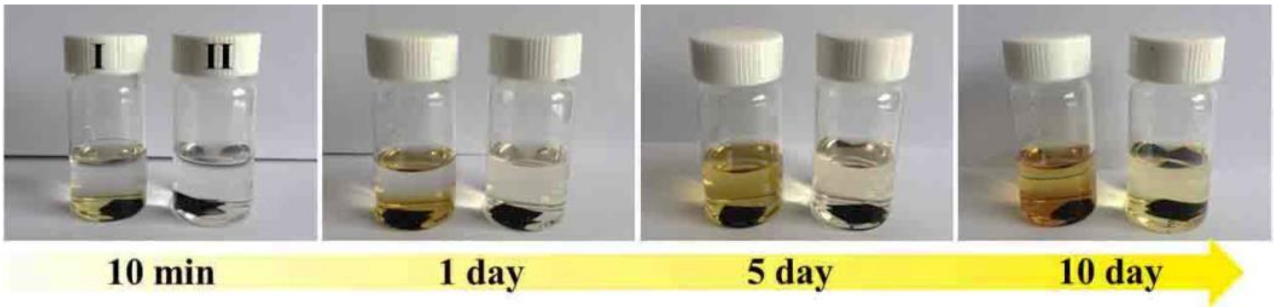

Supplementary Figure 8. Solubility tests of iodine on the pure carbon cloth (I) and HPCM-NP (II) (iodine content: 2 mg cm^{-2}).

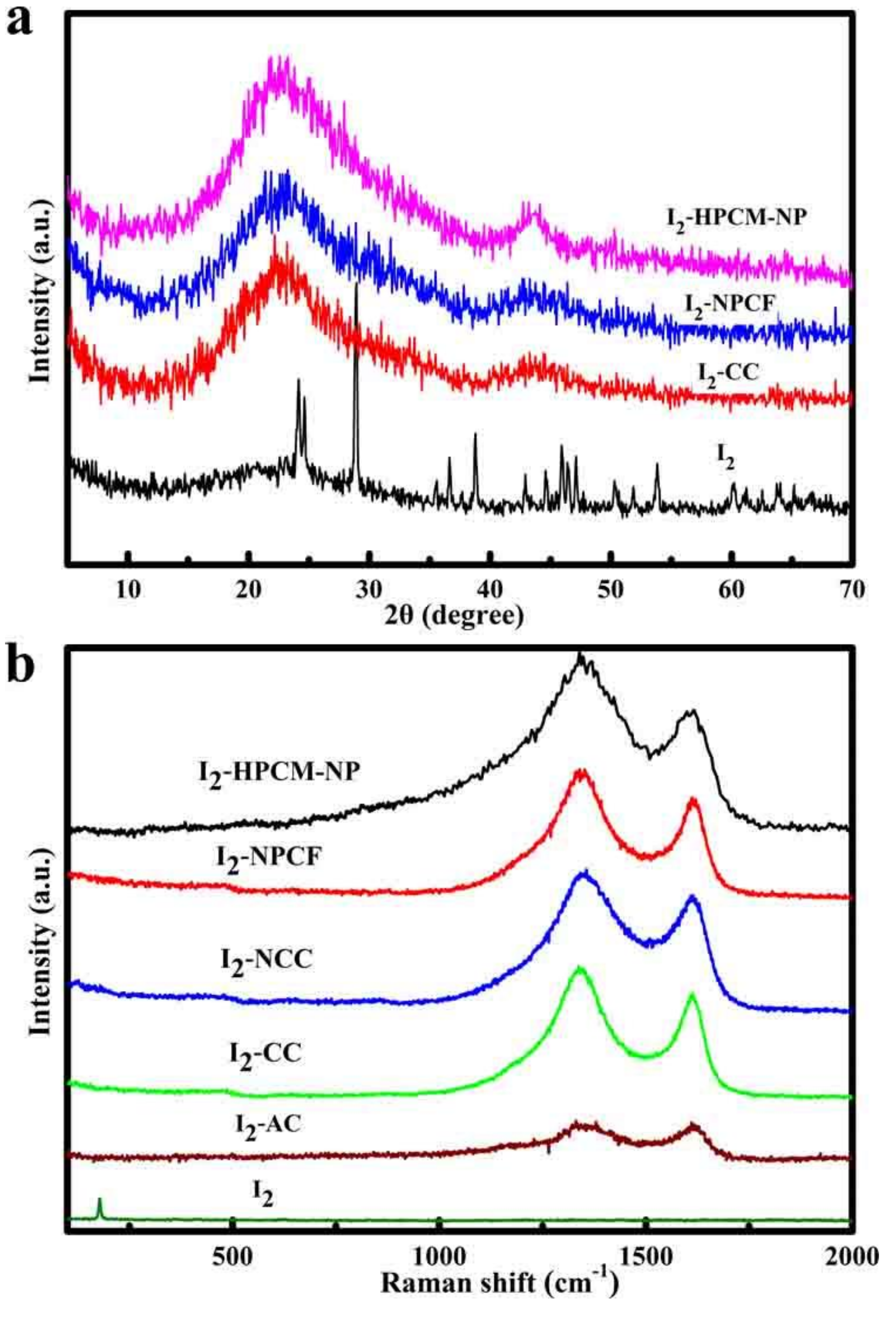

Supplementary Figure 9. (a) XRD patterns and (b) Raman spectra of various samples.

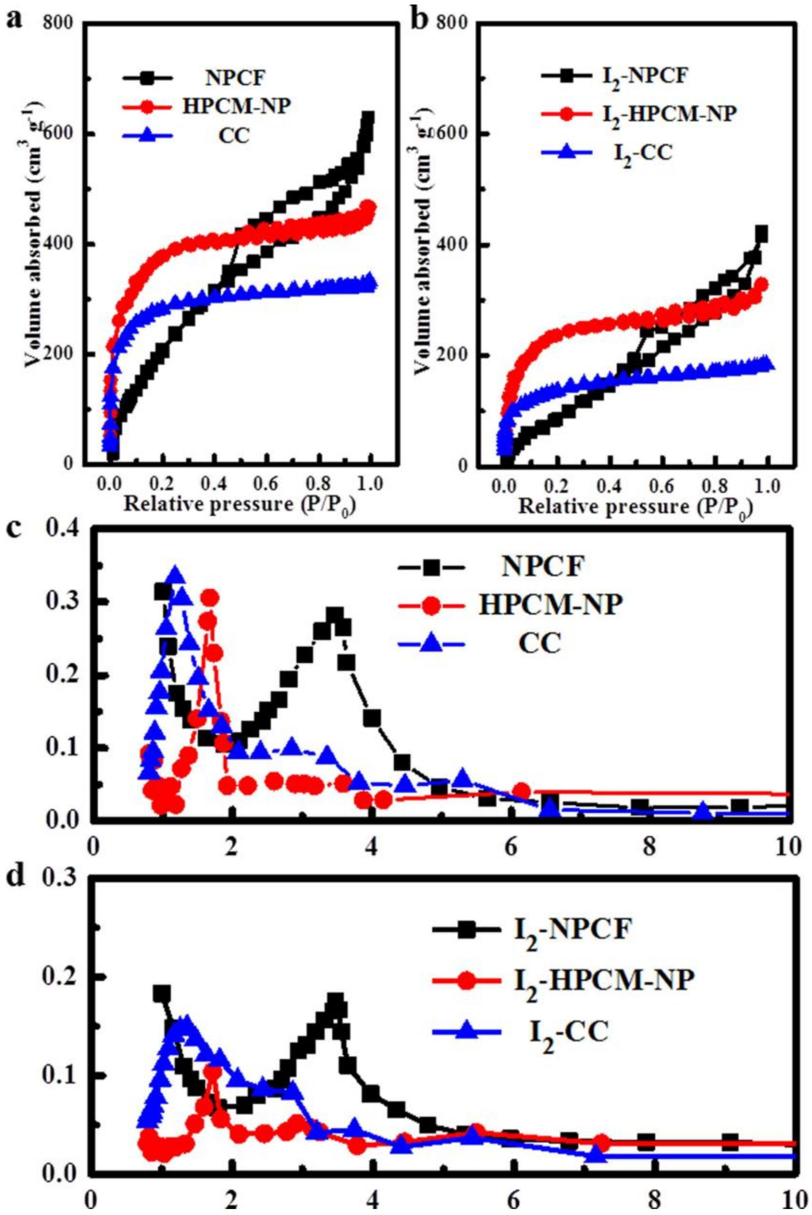

Supplementary Figure 10. (a) N_2 adsorption-desorption isotherms and (b) pore size distribution plots of iodine-carbon composites (iodine, 30 wt%).

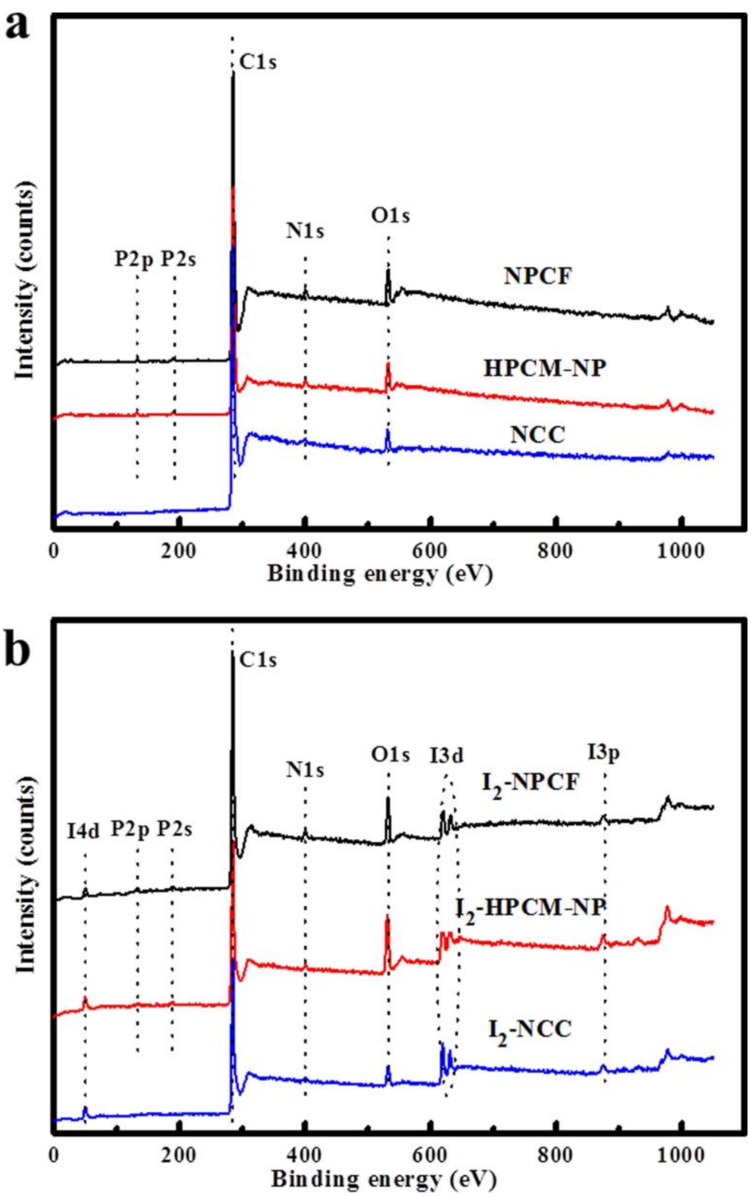

Supplementary Figure 11. XPS survey spectra of carbon samples (a) before and (b) after loading of iodine.

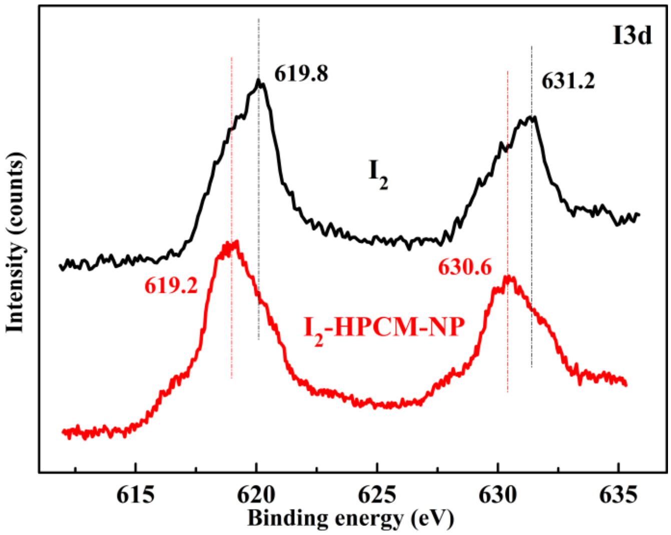

Supplementary Figure 12. High-resolution XPS spectra of I_{3d} for I_2 and I_2-HPCM-NP.

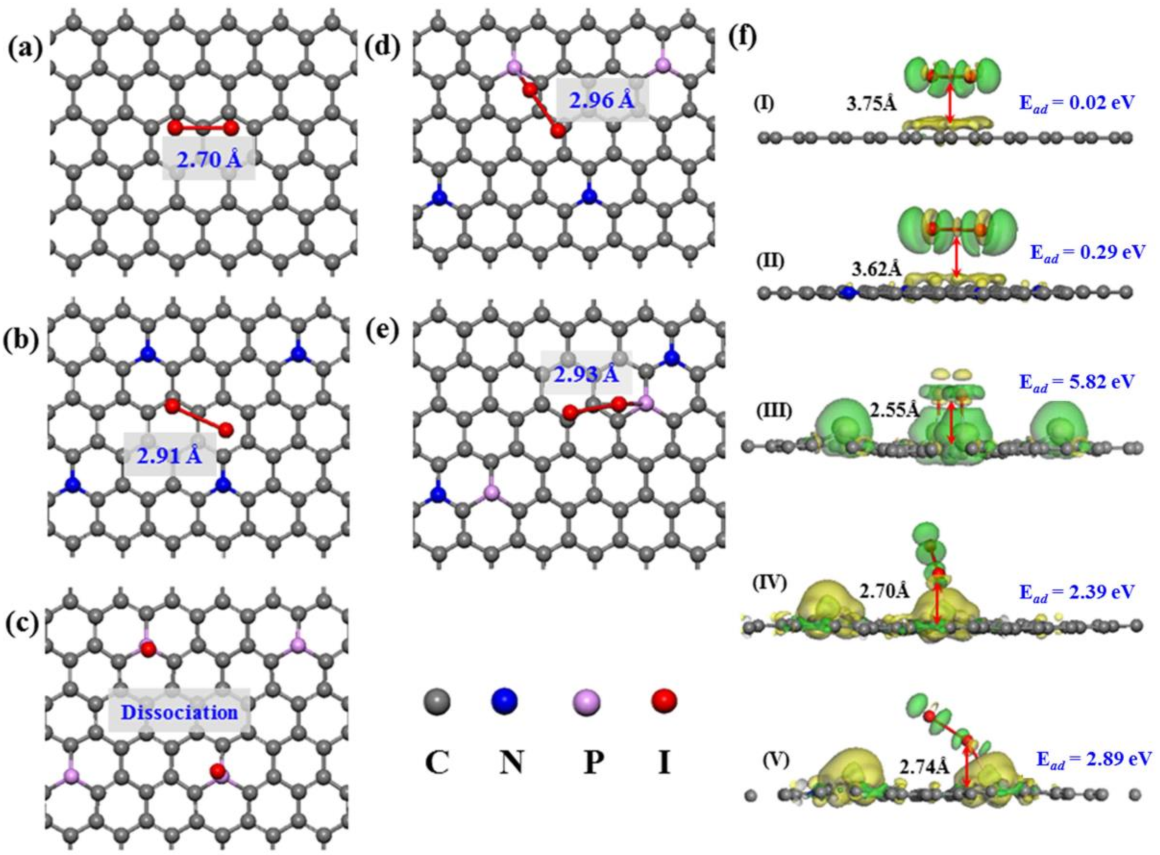

Supplementary Figure 13. The schematic illustration for the optimized configurations of iodine adsorped on the surface of the pristine graphene (a), N-doped graphene (b), P-doped graphene (c), isolated N and P (d), and coupled N and P (e) co-doped graphene, respectively. The contour plots of the different charge densities for the optimized structures of iodine molecule adsorbed on graphene (I), graphene doped with N (II), P (III), isolated N and P (IV), and coupled N and P (V). The differential charge density was calculated from: $\Delta\rho = \rho_{12} - \rho_1 - \rho_2$, where ρ_{12} and ρ_1 are the chare density of graphene and doped graphene with and without iodine adsorbed on the surface, respectively. Yellow and green color indicated the charge depletion and accumulation, respectively. The adsorption energies for I_2 molecule on these fragments were obtained using: $E_{ad} = -(E_1 + E_2 - E_{12})$, where E_{ad} is the adsorption energy of the I_2 molecule on the corresponding surfaces, E_1 is the total energy of the graphene (or heteroatom-doped graphene), E_2 is the energy of one isolated I_2 molecule, and E_{12} is the energy of the optimized structures for I_2 molecules adsorbed on the graphene planes. The bonds length of adsorbed I_2 molecule and the vertical distance of I_2 molecule to the substrate are also listed in the figure, along with the adsorption energies.

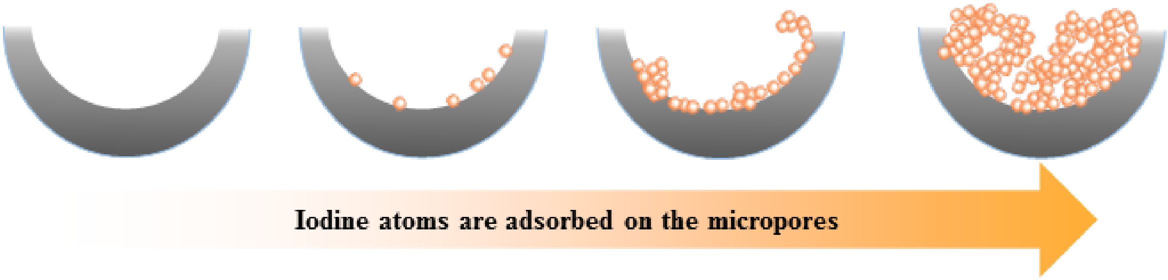

Supplementary Figure 14. Schematic illustration for the proposed adsorption process of iodine.

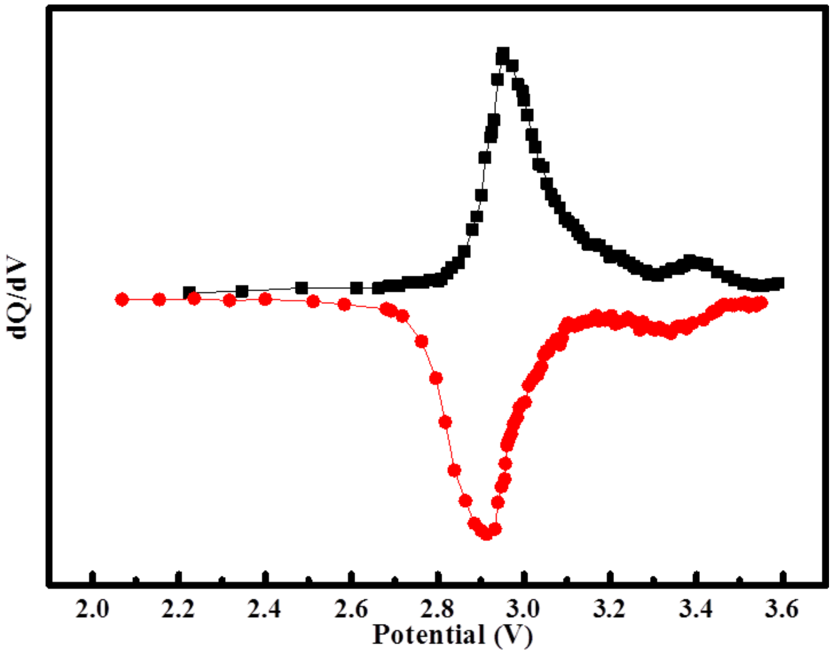

Supplementary Figure 15. Differential capacity plots of Li-iodine (I_2-HPCM-NP//Li) batteries.

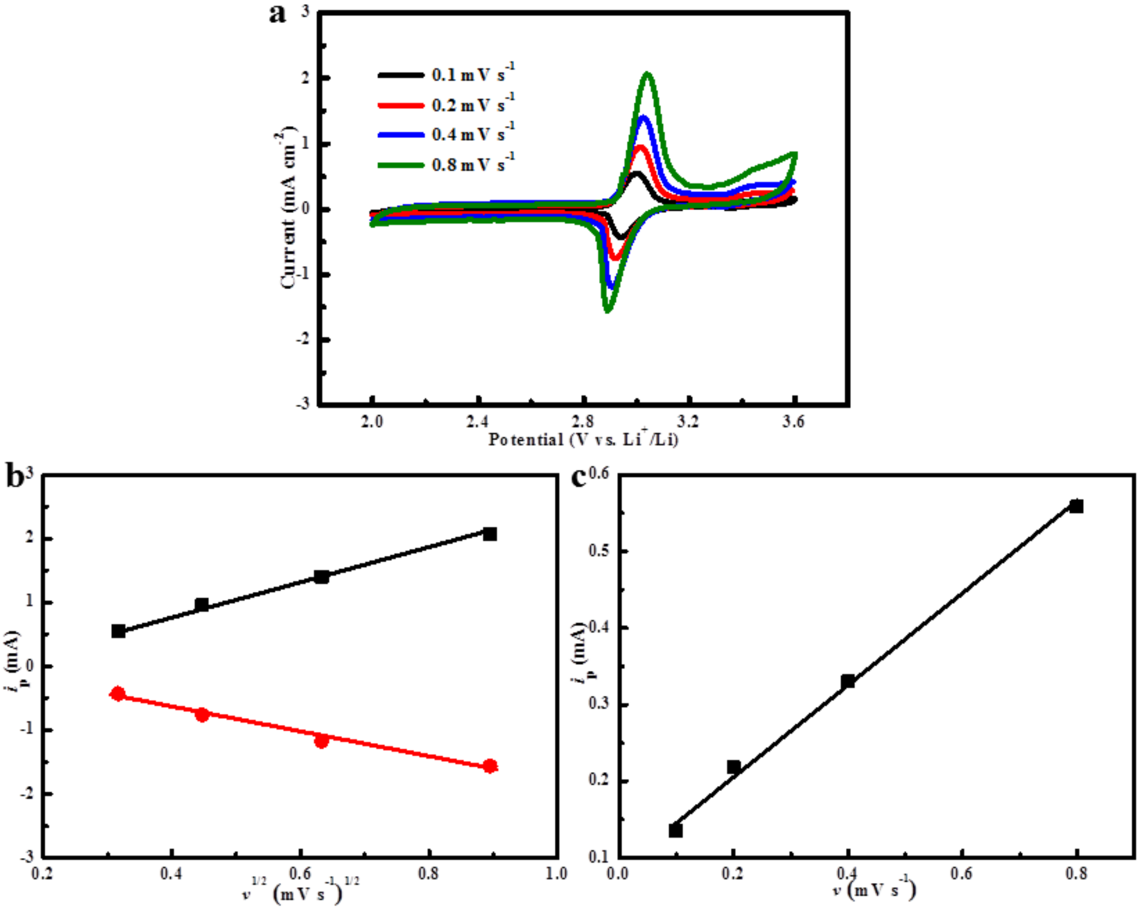

Supplementary Figure 16. (a) CVs of the I_2-HPCM-NP electrode at different scan rates. The relationships between the peak current (b, lower peak; c, higher peak) and scan rate in the cathodic/anodic process, respectively.

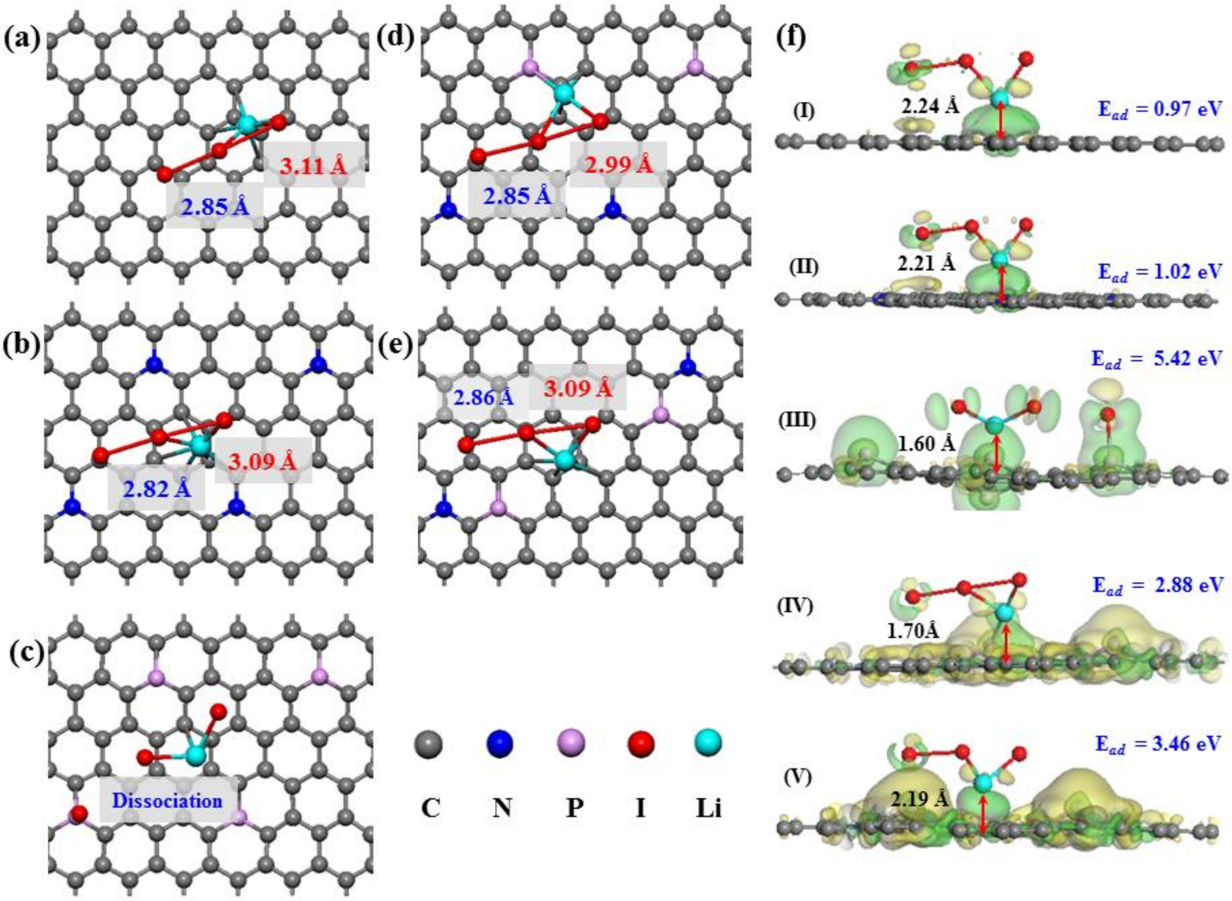

Supplementary Figure 17. Schematic illustration for the optimized configurations of LiI_3 adsorbed on the surface of the pristine graphene (a), N-doped graphene (b), P-doped graphene (c), isolated N and P (d), and coupled N and P (e) co-doped graphene, respectively. The contour plots of the different charge densities for the optimized structures of LiI_3 adsorbed on graphene (I), graphene doped with N (II), P (III), isolated N and P (IV), and coupled N and P (V). The differential charge density was calculated: $\Delta\rho = \rho_{12} - \rho_1 - \rho_2$, where ρ_{12} and ρ_1 are the chare density of graphene and doped graphene with and without LiI_3 adsorbed on the surface, respectively. ρ_2 is the chare density of LiI_3. Yellow and green color indicate the charge depletion and accumulation, respectively. The adsorption energies for LiI_3 on these fragments were obtained: $E_{ad} = -(E_1 + E_2 - E_{12})$, where E_{ad} is the adsorption energy of LiI_3 on the corresponding surfaces, E_1 is the total energy of the graphene (or heteroatom doped graphene), E_2 is the energy of isolated LiI_3, and E_{12} is the energy of the optimized structures for LiI_3 adsorbed on the graphene planes. The bond lengths of I-I in LiI_3 and vertical distance from the adsorbed LiI_3 to the substrate are also listed in the figure, along with the adsorption energies.

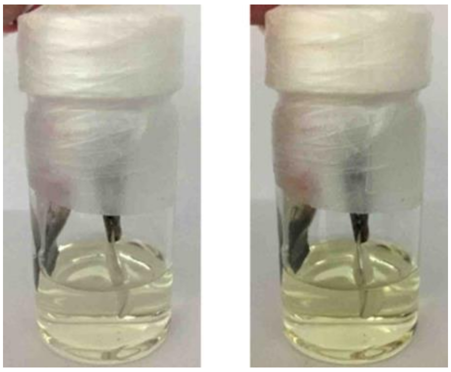

Supplementary Figure 18. Photographs of Li-I_2 batteries before (left) and after (right) the cycling stability test (iodine content: 1.5 mg cm^{-2}).

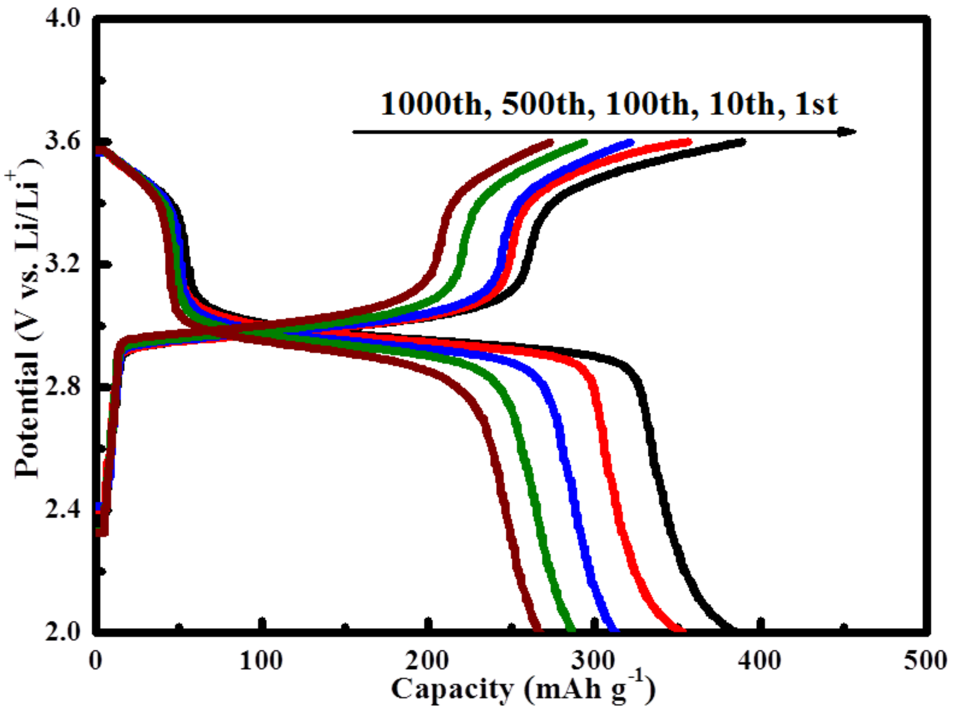

Supplementary Figure 19. Typical charge/discharge curves of Li-iodine (I_2-HPCM-NP//Li) batteries.

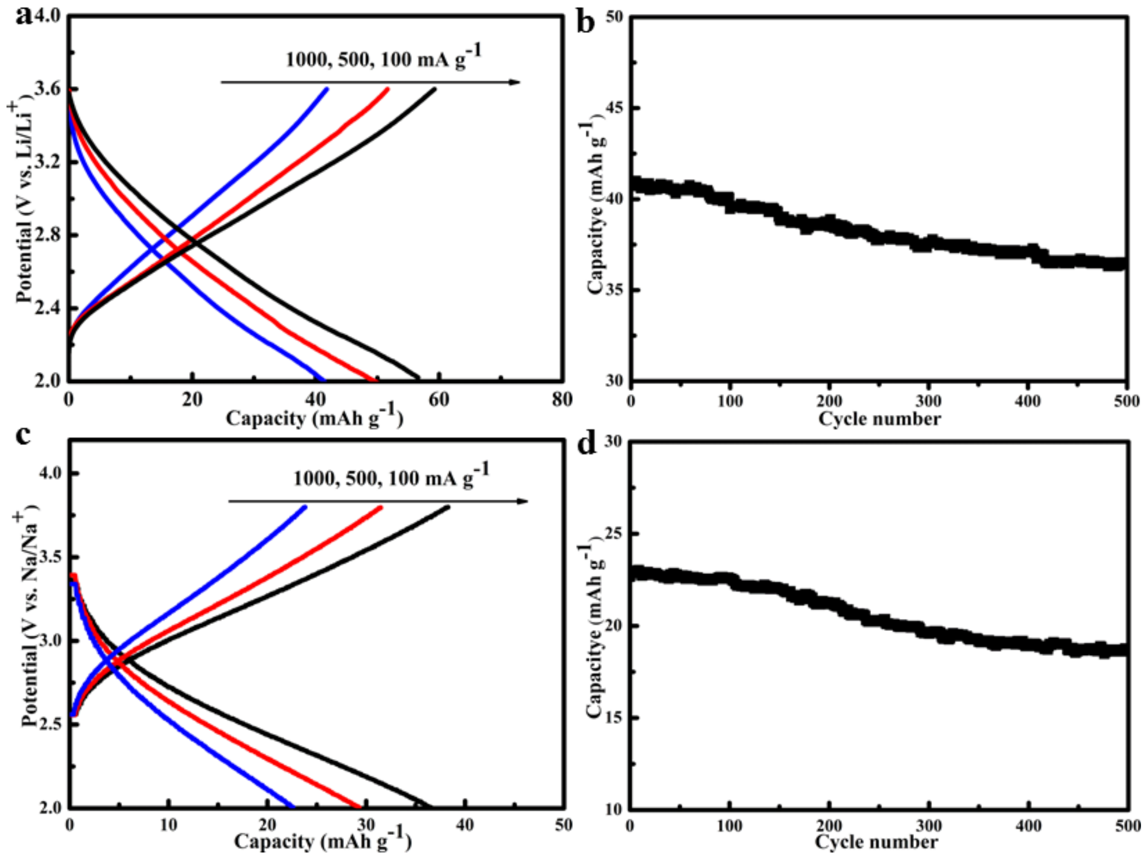

Supplementary Figure 20. (a, c) Charge/discharge curves and (b, d) long-term cycling stability tests of HPCM-NP for Li and Na storage.

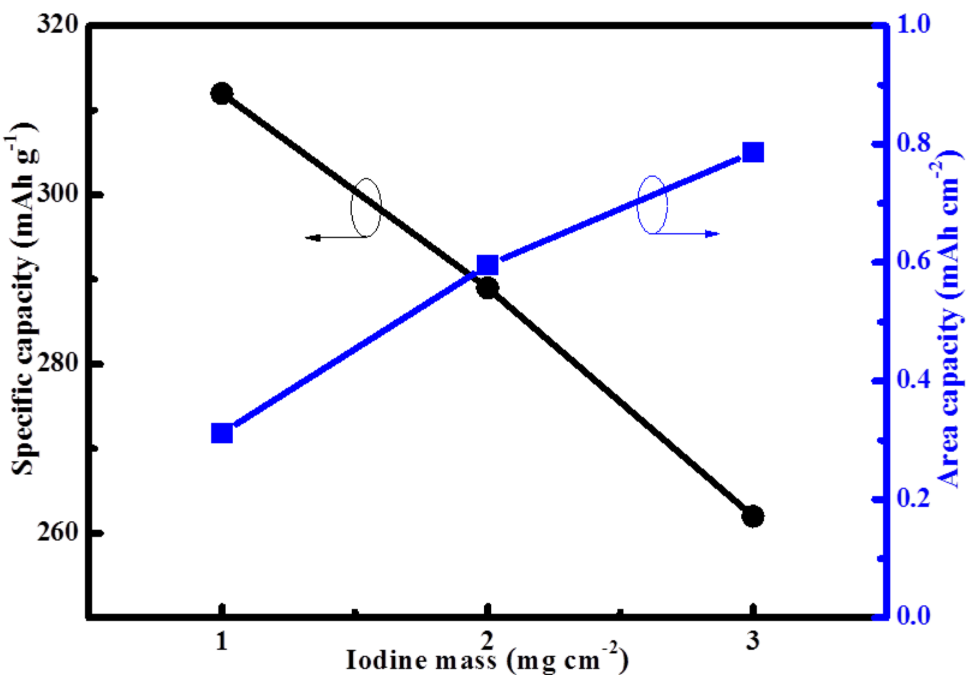

Supplementary Figure 21. (a) Initial discharge capacities of iodine-carbon composite cathodes with various iodine loadings.

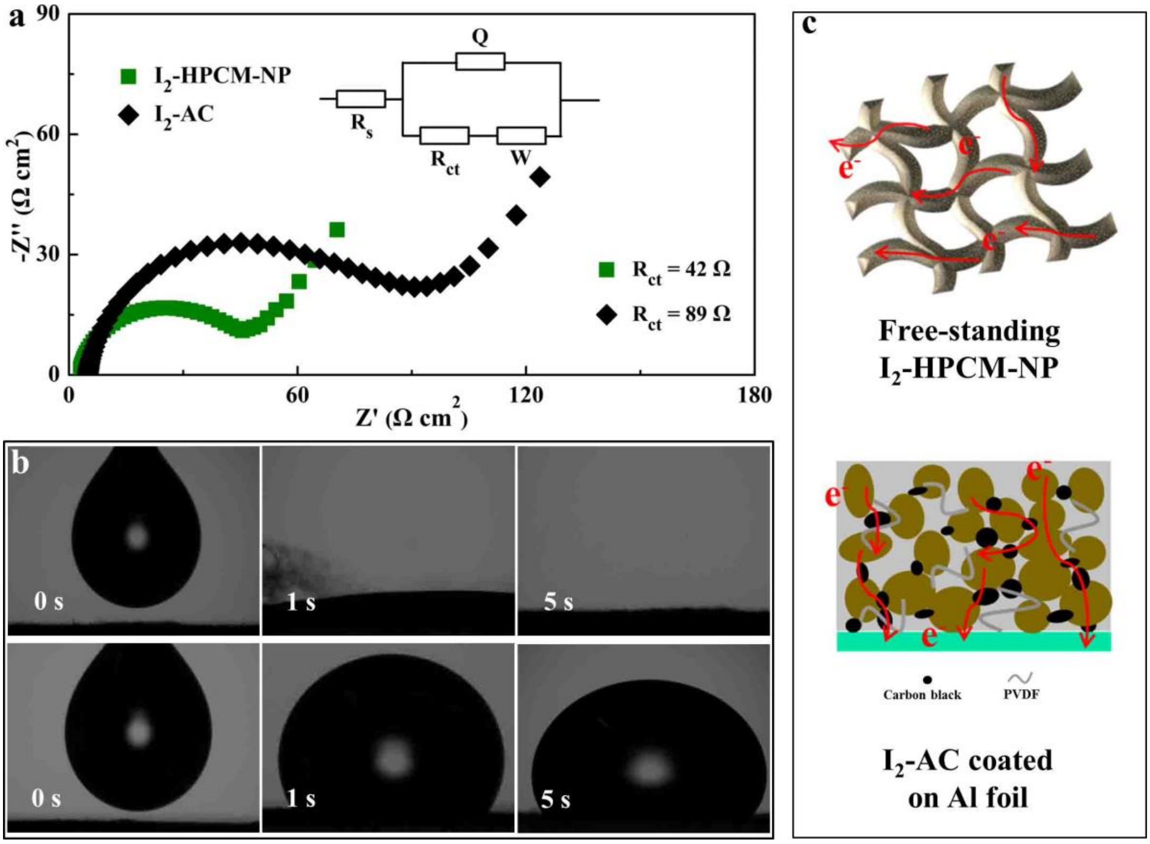

Supplementary Figure 22. (a) Nyquist plots for the I_2-HPCM-NP and I_2-AC electrodes. Inset is a simplified equivalent circuit. (b) Photographs of electrolyte droplet placed onto the surface of I_2-HPCM-NP electrode (up) and I_2-AC electrode (down), respectively. (c) Schematic illustration for the continuous electron pathway of the I_2-HPCM-NP electrode compared with that of the I_2-AC electrode.

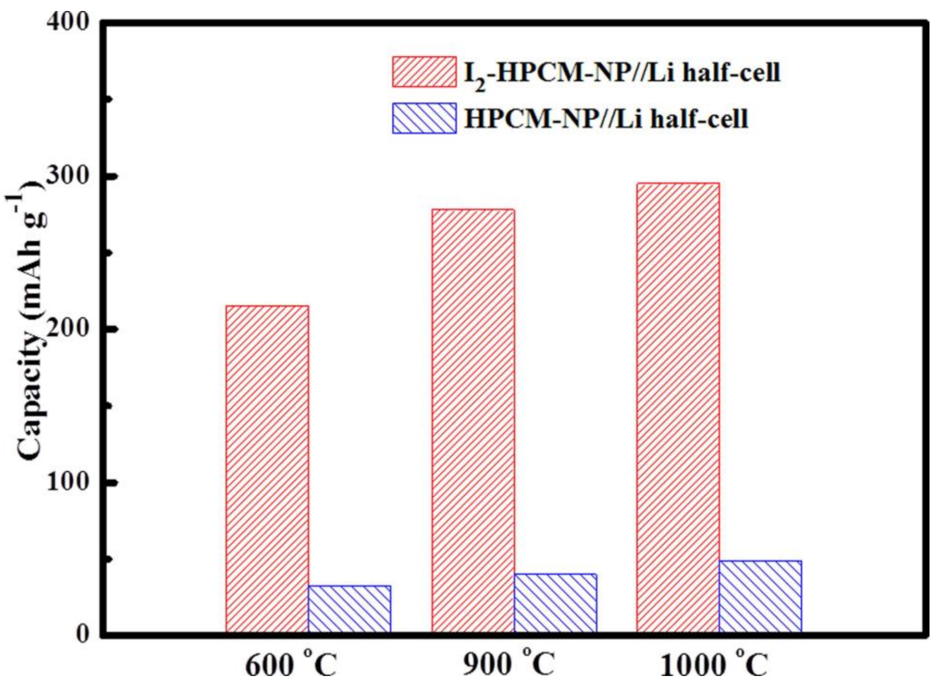

Supplementary Figure 23. Specific capacity of batteries using HPCM-NP and I_2-HPCM-NP (between 2.0 and 3.6 V vs. Li^+/Li) prepared at different temperatures, respectively.

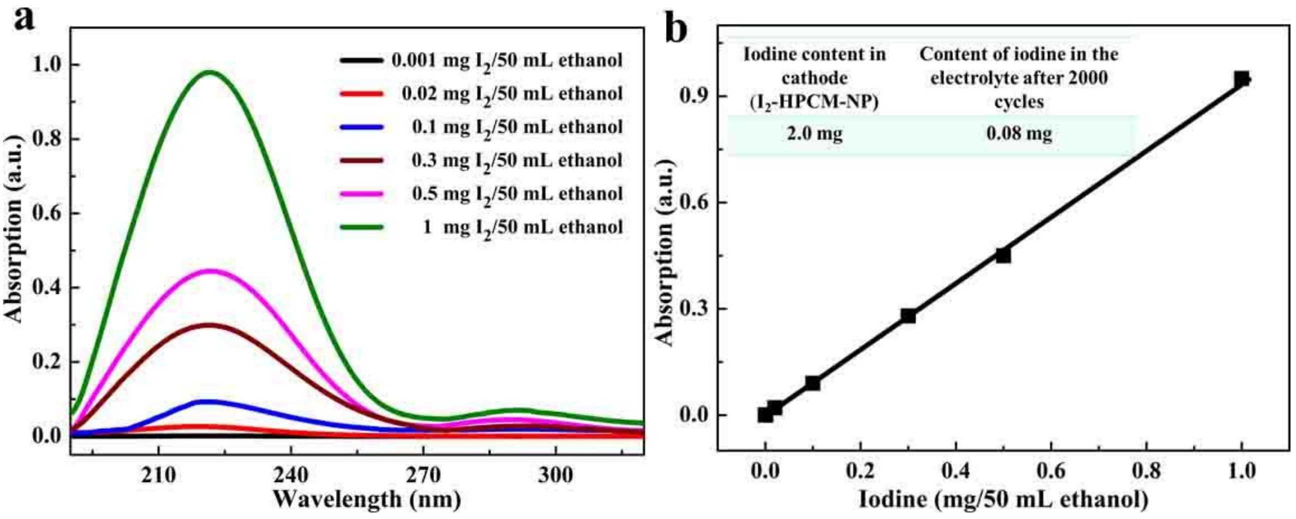

Supplementary Figure 24. (a) The typical absorption spectra of iodine dissolved in ethanol with different concentrations. (b) The linear relationship between the UV absorbance and the concentrations of iodine. Inset shows the mass loading of iodine in HPCM-NP electrode and the content of iodine in the electrolyte measured after 2000 cycles (current density: 500 mA g^{-1}).

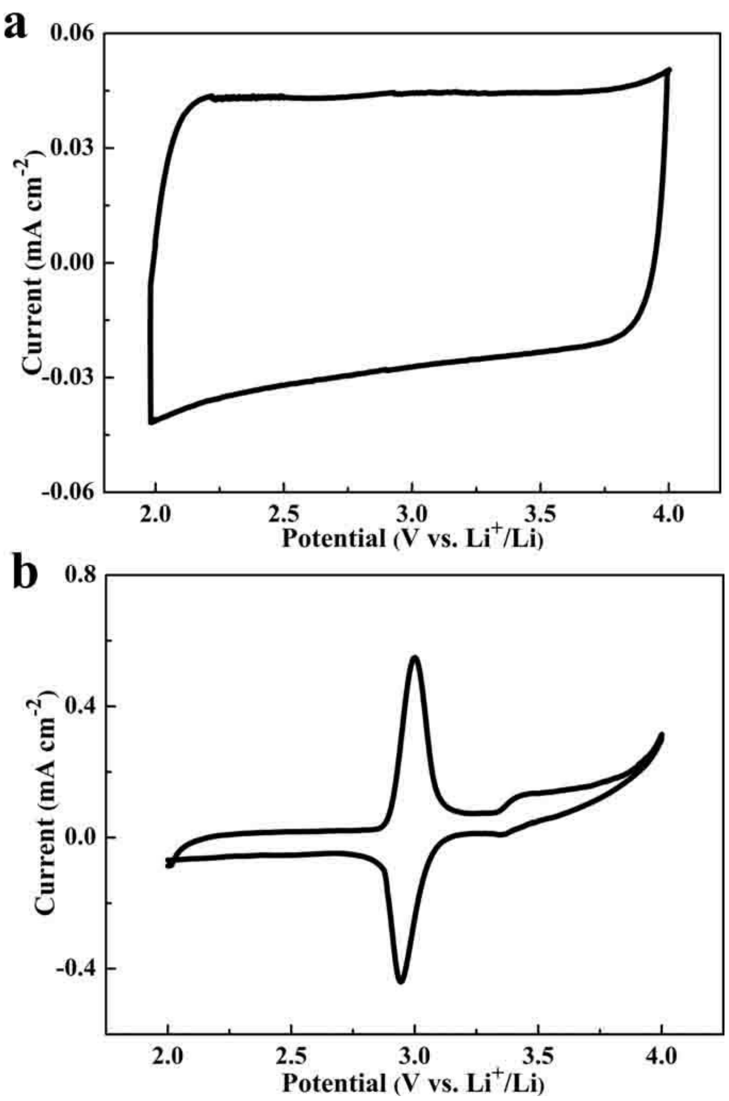

Supplementary Figure 25. Cyclic voltammogram profiles of HPCM-NP (a) and I_2-HPCM-NP (b) electrodes at a scan rate of 0.1 mV s^{-1} in the potential range of 2.0 ~ 4.0 V, respectively.

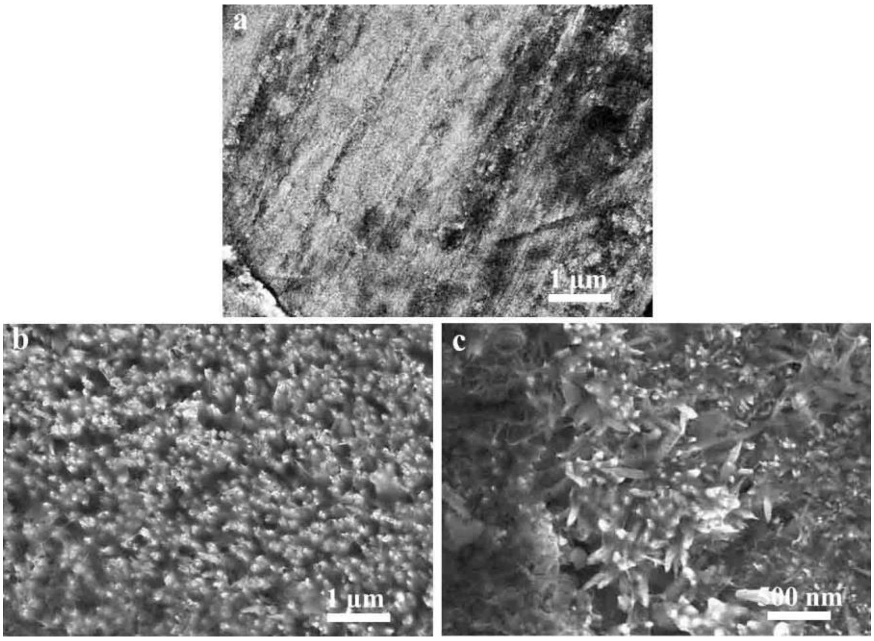

Supplementary Figure 26. SEM images of the Li-metal electrode (a) before and (b, c) after 2000 charge/discharge cycles.

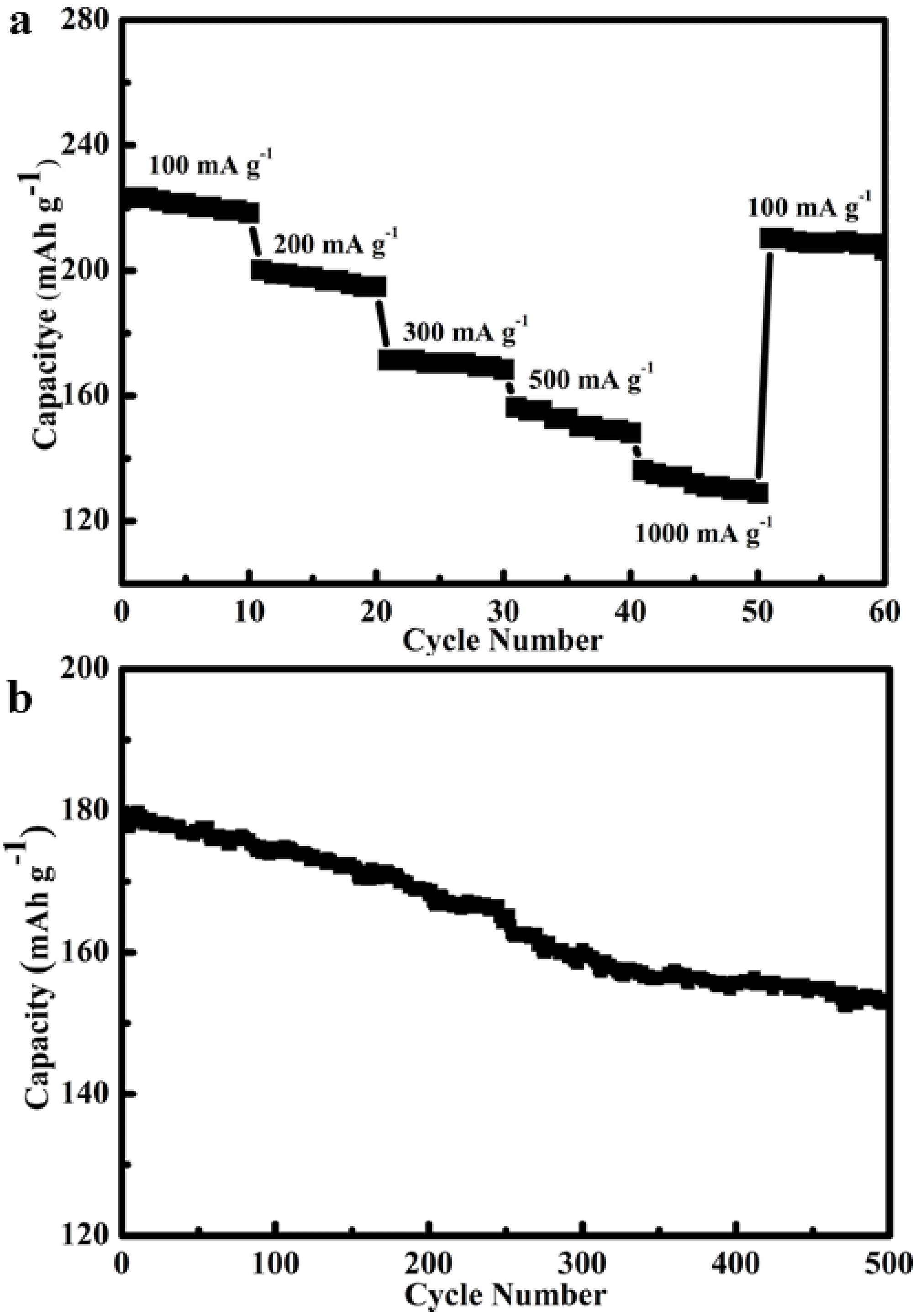

Supplementary Figure 27. (a) Rate capability and (b) cycling stability test of Na-iodine batteries.

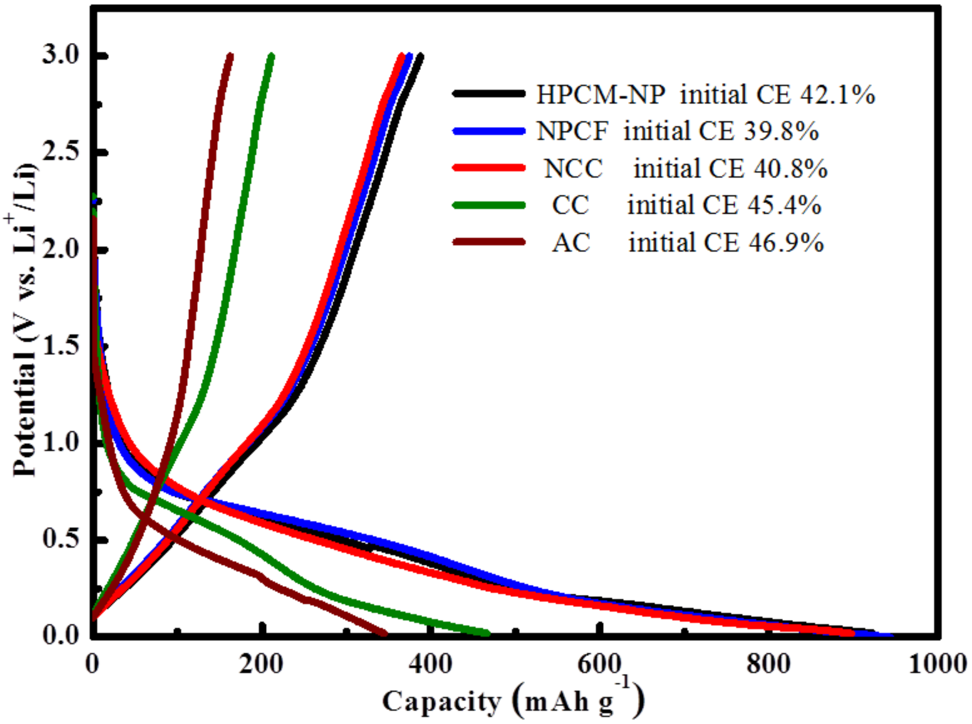

Supplementary Figure 28. Initial discharge and charge profiles of different carbon anodes for LIBs.

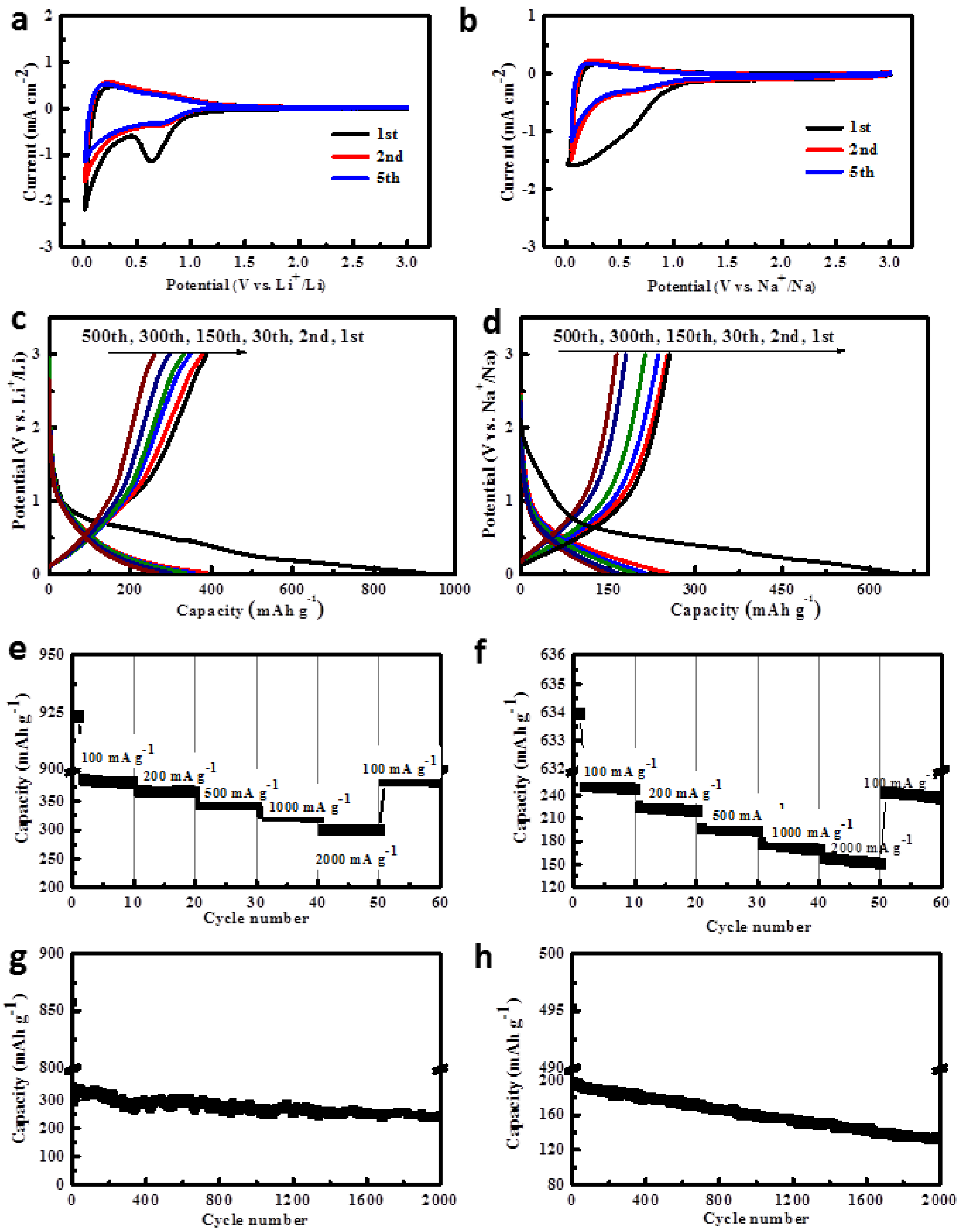

Supplementary Figure 29. CV curves of HPCM-NP anode for (a) LIBs and (b) NIBs. Discharge/charge curves of HPCM-NP anode for (c) LIBs and (d) NIBs at low-current density of 100 mA g^{-1}. Rate capabilities of HPCM-NP anode for (e) LIBs and (f) NIBs. Cycling stabilities of HPCM-NP anode for (g) LIBs and (h) NIBs.

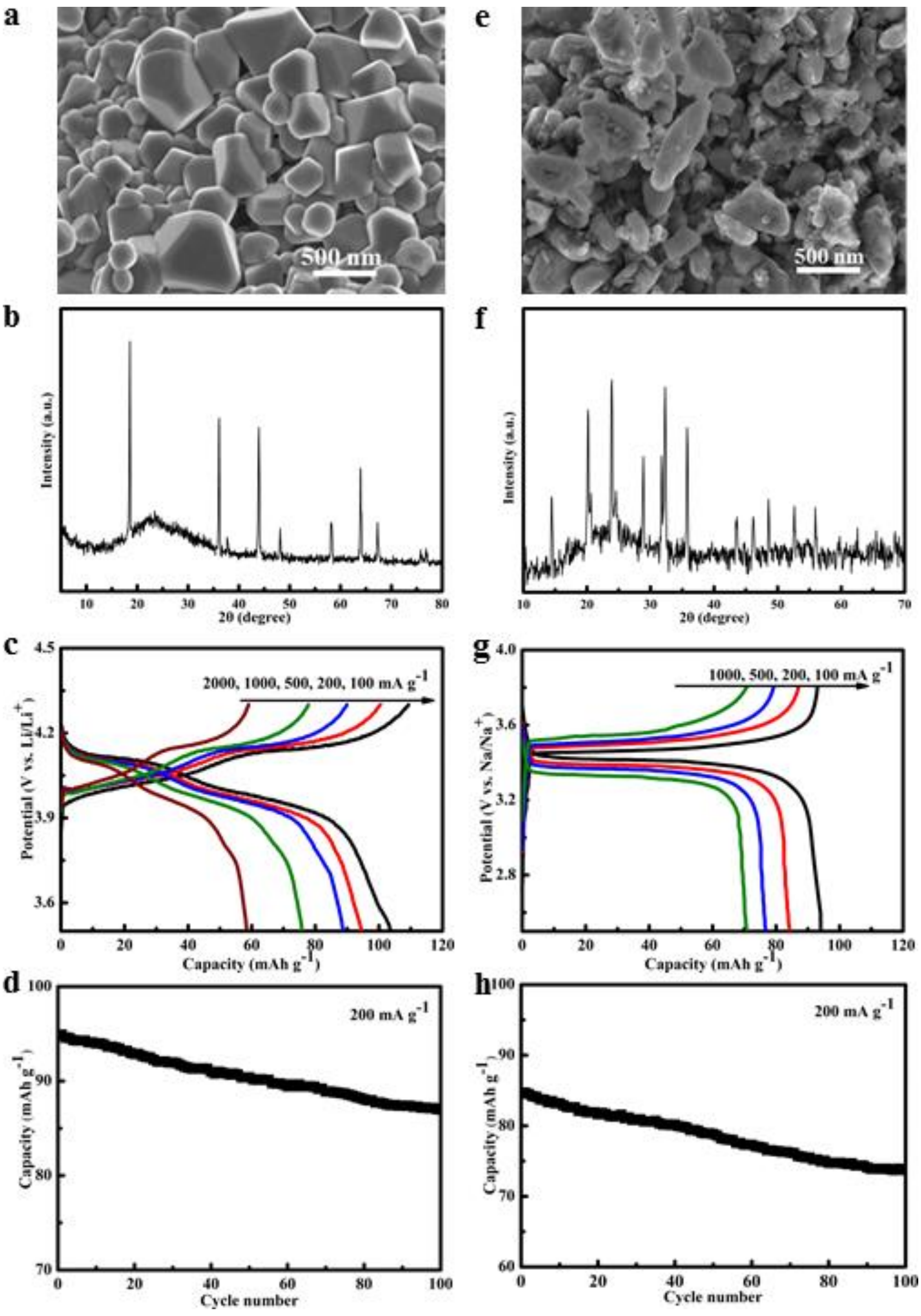

Supplementary Figure 30. (a) SEM image and (b) XRD pattern of $LiMn_2O_4$ power. (c) Discharge/charge curves and (d) cycling performance of $LiMn_2O_4$ as LIBs cathode. (e) SEM image and (f) XRD pattern of $Na_3V_2(PO_4)_3/C$. (g) Discharge/charge curves and (h) cycling performance of $Na_3V_2(PO_4)_3/C$ as NIBs cathode.

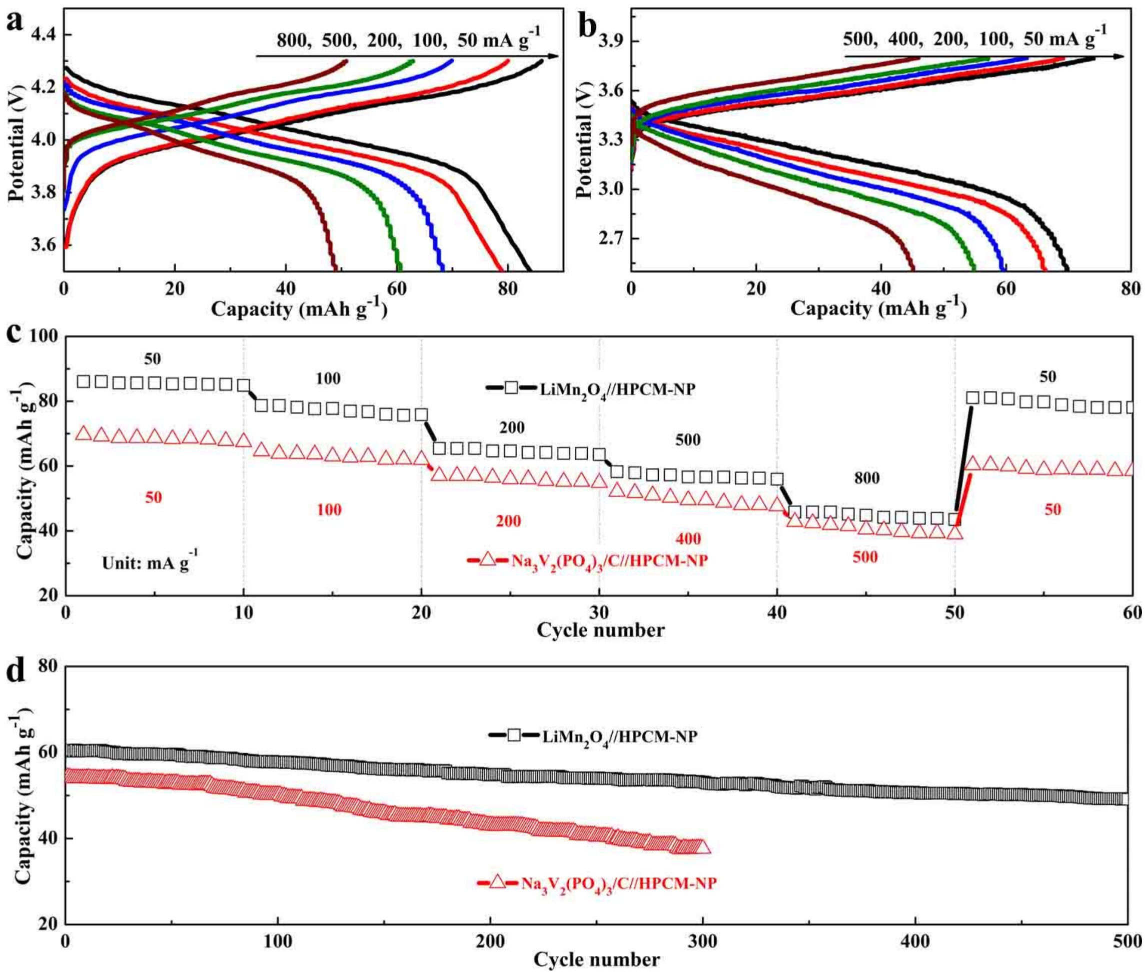

Supplementary Figure 31. Charge-discharge profiles of (a) Li-ion and (b) Na-ion full cells with $LiMn_2O_4$ and $Na_3V_2(PO_4)_3/C$ as cathodes, respectively, and the corresponding discharge capacities (up: based on the weight of anode and cathode; down: based on the weight of carbon anode). (c) Rate capability and (d) cycling performance of Li-ion and Na-ion full cells.

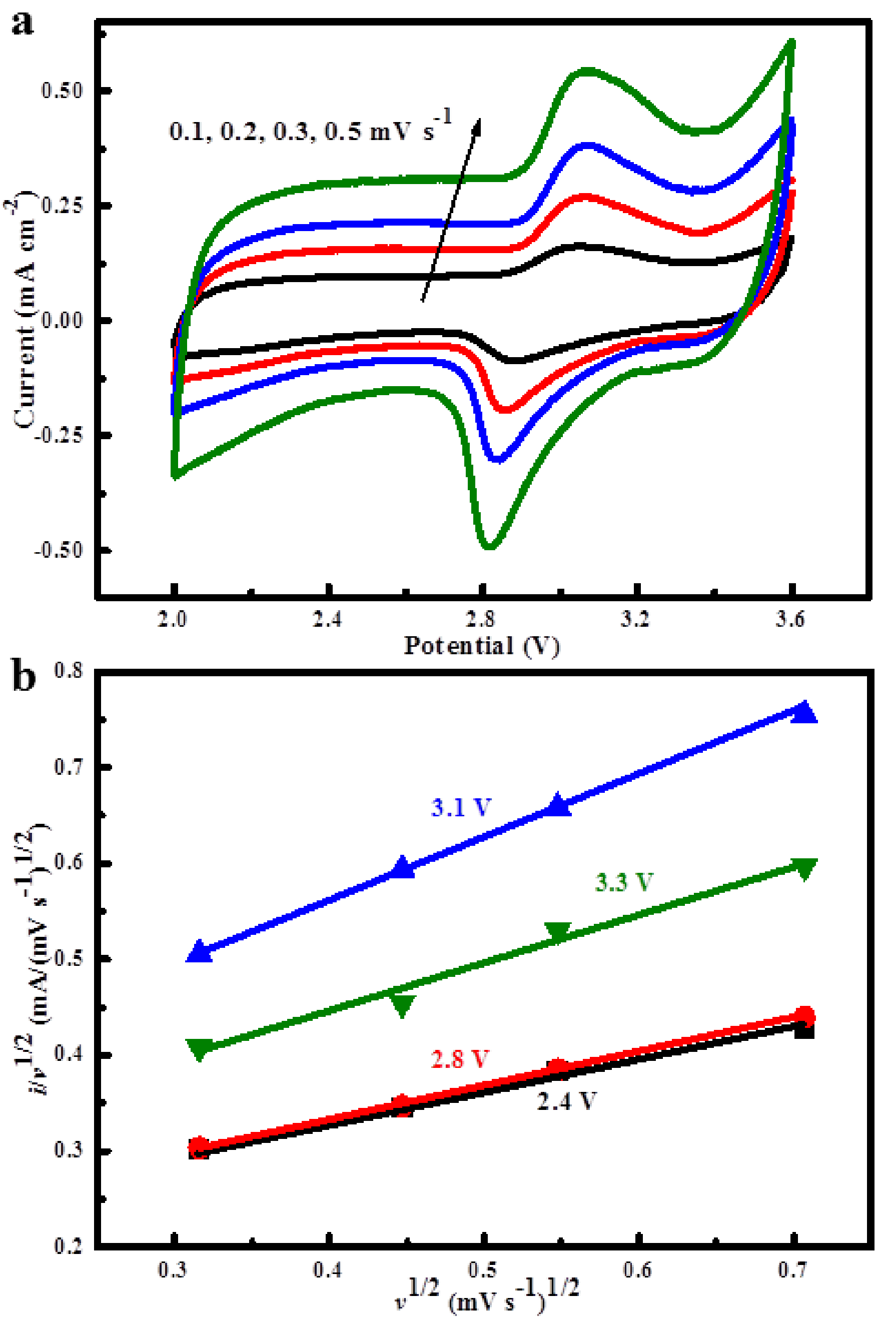

Supplementary Figure 32. (a) CV curves of I_2-HPCM-NP// HPCM-NP full battery using a Li-ion electrolyte at different scan rates. (b) Plots of $v^{1/2}$ vs. $i/v^{1/2}$ at different potentials. Here, v (mV s^{-1}) and i (mA) are scan rate and current values at different potentials, respectively.

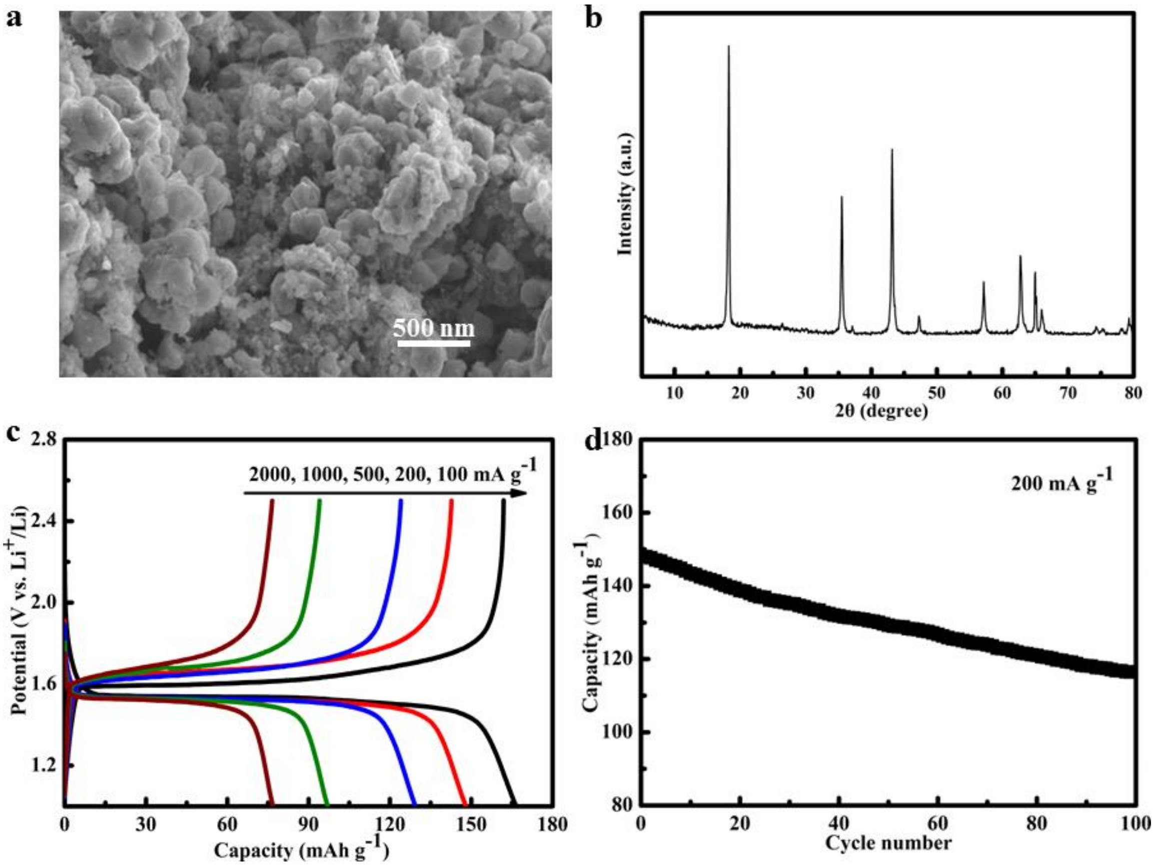

Supplementary Figure 33. (a) SEM image and (b) XRD pattern of $Li_4Ti_5O_{12}$ power. (c) Discharge/charge curves and (d) cycling performance of $Li_4Ti_5O_{12}$ as LIBs anode.

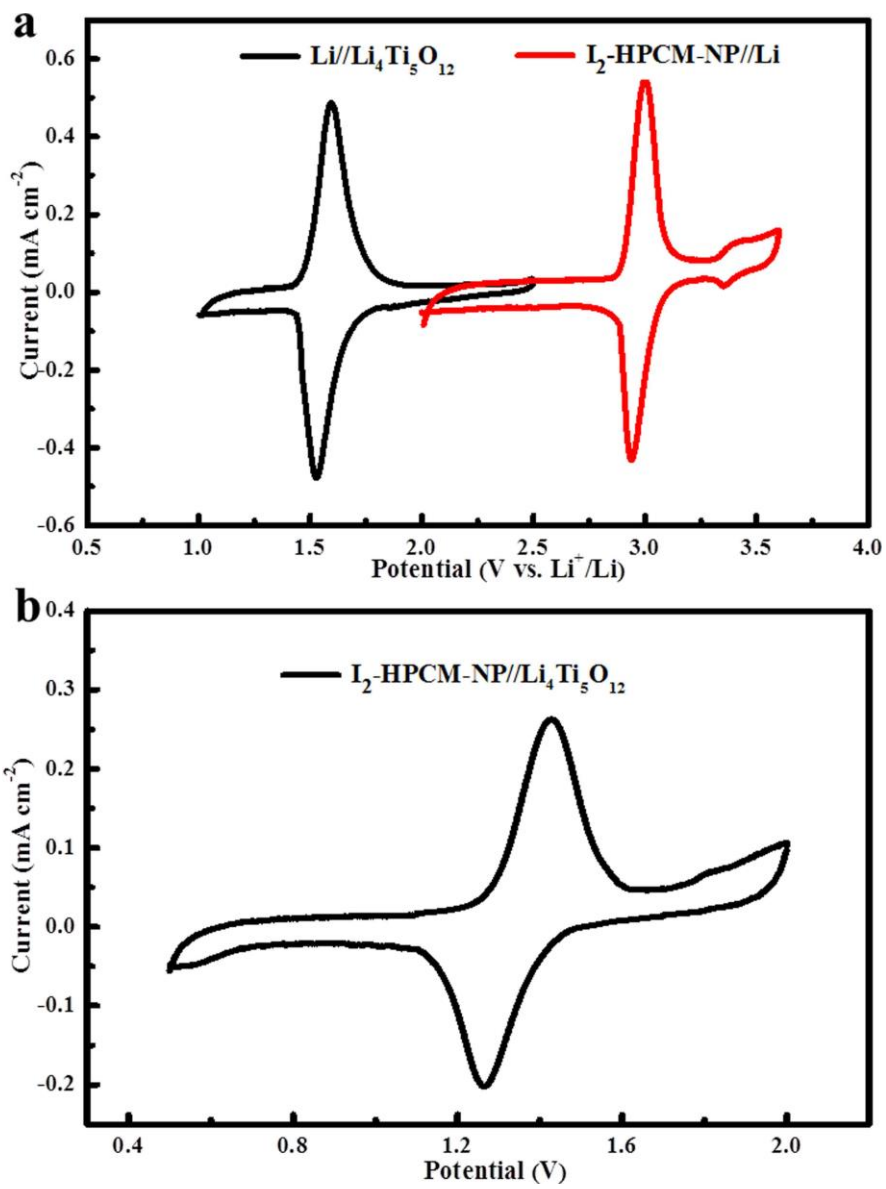

Supplementary Figure 34. CV curves of (a) I_2-HPCM-NP, commercial $Li_4Ti_5O_{12}$, and (b) I_2-HPCM-NP//$Li_4Ti_5O_{12}$ full cell at a scan rate of 0.1 mV s^{-1}.

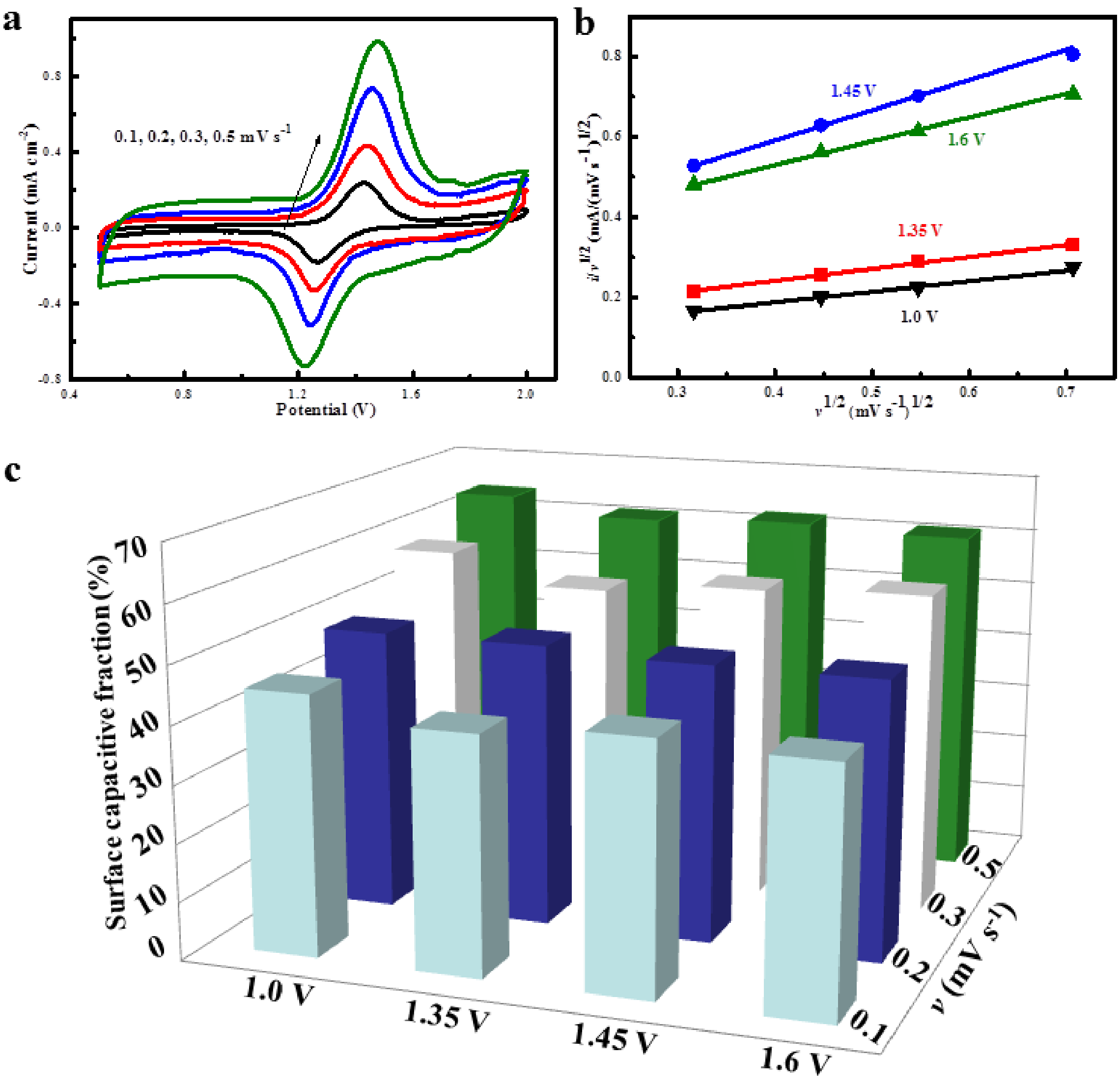

Supplementary Figure 35. (a) CV curves of I_2-HPCM-NP//$Li_4Ti_5O_{12}$ full battery using a Li-ion electrolyte at different scan rates. (b) Plots of $v^{1/2}$ vs. $i/v^{1/2}$ at different potentials and (c) Surface capacitive contribution (k_1v) at a specific potential according to the Equation ($i = k_1v + k_2v^{1/2}$). Here, v (mV s^{-1}) and i (mA) are scan rate and current values at different potentials, respectively. k_1 and k_2 were determined by plotting the scan rate against the current at different potentials.

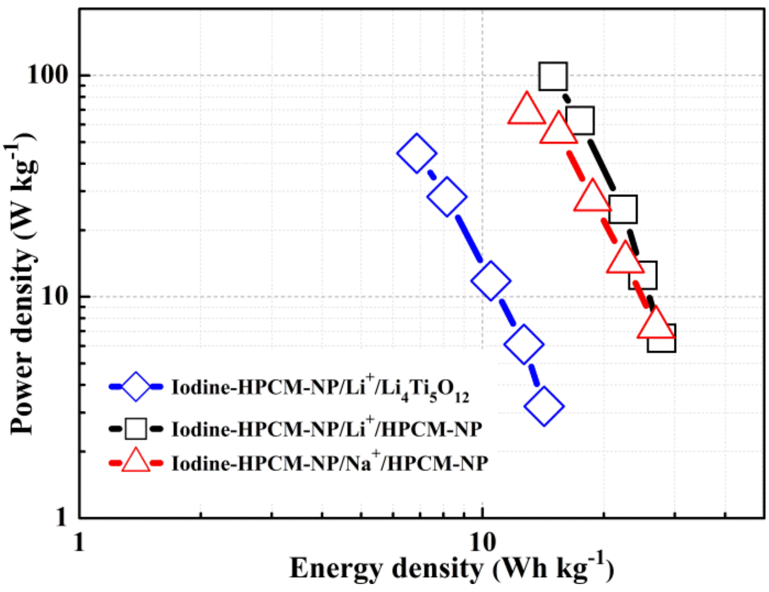

Supplementary Figure 36. Ragone plot of full batteries, where power and energy densities were estimated based on the total weight of cathode material, anode material and electrolyte.

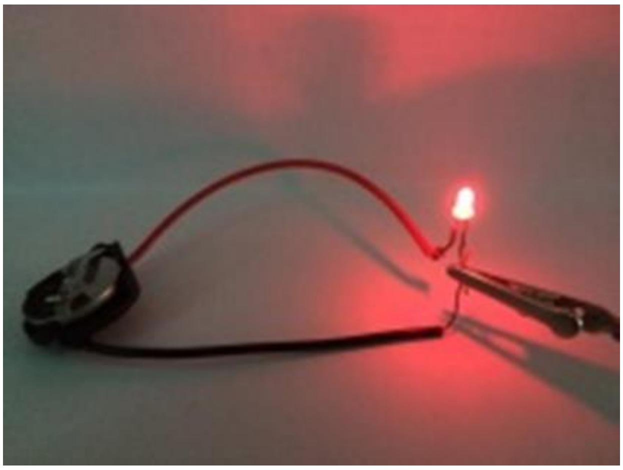

Supplementary Figure 37. Optical images of a red LED being driven by the iodine-carbon hybrid full battery.

Supplementary Table 1. Electrochemical performances of Li-iodine batteries.

Matrix	Capacity (mAh g^{-1})/ current density (mA g^{-1})	Capacity retention (%)/ cycles	Ref.
Solid-state Li-I_2thin film battery	49.6 μAh cm^{-2}/6 μA cm^{-2}	~25/5	1
B_2O_3-modified carbon microtubes	200/20C	~76.8/5000	2
Carbon black	300/101	71.4/1000	3
Carbon cloth	301/105	64.8/300	4
HPCM-NP	386/100	84.5/2000	This work

Supplementary Table 2. Overview of carbon anodes for Li and Na ion batteries (LIBs and NIBs).

Type of carbon	Capacity (mAh g^{-1})/ current density (mA g^{-1})	Capacity retention (%)/ cycles	Ref.
	LIBs		
Porous carbon sphere	378/37.2	96.6/100	5
Coconut kernel derived carbon	195/728	81.3/50	6
Carbon aerogels	288/375	93/100	7
N-porous carbon	551/100	94/100	8
Carbonized lignin	164.9/200	109/400	9
HPCM-NP	386/100	70.9/2000	This work
	NIBs		
Hard Carbon	240/25	91.7/100	10
Hollow Carbon Nanowires	251/50	82.2/400	11
N-porous carbon nanosheets	349.7/50	44.4/260	12
N-doped porous carbon fibers	296/50	~88/600	13
Carbon Nanofiber	233/50	97.7/200	14
N-doped carbon nanofibers	134.2/200	88.7/200	15
HPCM-NP	252/100	67.7/2000	This work

Supplementary Note 1. Detailed discussion on the effect of synthetic parameters on materials properties.

HPCM-NP samples have been synthesized by changing the ratios of aniline to phytic acid (Supplementary Figs. 1 and 2). The effect of amount of aniline on the morphology evolution and thickness of the surface layer of the HPCM-NP is shown in Supplementary Fig. 1. It can be seen that polyaniline was uniformly deposited on the carbon fiber matrix, and the thickness of the carbon layer increased with increasing the aniline monomers from 0.8 to 1.2 mL. However, further increasing the aniline monomers resulted in the formation of free-standing polyaniline macro-particles randomly attached on the surface (Supplementary Fig. 1d), but did not obviously increase the thickness of porous carbon film due to the limited binding affinity to the core fiber. The surface becomes uneven and the as-deposited polyaniline hydrogels were easily detached from carbon fibers during the drying process. The nitrogen adsorption-desorption isotherm (Supplementary Fig. 2a,b) revealed the gradual formation of highly porous structure with a large surface area. Typically, the specific surface area increased from ~673, 1182 to 1487 $m^2 g^{-1}$ for the resultant samples named as HPCM-$NP_{0.8}$, HPCM-$NP_{1.0}$ and HPCM-$NP_{1.2}$. However, as the volume addition of aniline increased to 1.4 mL, the specific surface area decreased to ~906 $m^2 g^{-1}$. It has been revealed that phytic acid played a crucial role for the formation of porous polyaniline aerogel[16,17]. Thus, the HPCM-$NP_{1.2}$ synthesized under the optimized ratio of aniline to phytic acid ratio exhibited a relatively narrow pore size distribution and high surface area, which would be beneficial for improving the electrochemical performance.

The iodine uptake curve (Supplementary Fig. 2c) exhibited the maximum adsorption content of iodine for HPCM-$NP_{1.2}$ due to its large specific surface area and unique porous structure. The galvanic charge/discharge measurements exhibited the initial capacity of HPCM-$NP_{1.2}$ electrode around 324 mAh g^{-1}, which was the largest among all the electrode materials tested in this study. These results revealed that the highly porous HPCM-NP with a large surface area and unique porous structure can be prepared under the optimized condition, leading to the improved battery performance.

Supplementary Note 2. The solubility tests of iodine adsorped on different carbon samples.

Owing to the unique porous structural feature and the desirable chemical composition (heteroatom doping), the HPCM-NP material exhibited an enhanced stability for iodine adsorption and a good long-term cycling stability for Li-I_2 batteries. As demonstrated in Supplementary Fig. 8, the adsorbed iodine on the carbon scaffold without heteroatoms doping was quickly dissolved in carbonate and ether solvent with dark brown color. However, the HPCM-NP solution showed only a slight brown color even after 10 days, indicating the adsorbed iodine has a strong interaction with the matrix. Thus, the HPCM-NP exhibited a much better stability for iodine adsorption in comparison with the pure carbon fiber.

Supplementary Note 3. XPS spectrum of I_{3d} for I_2-HPCM-NP.

XPS spectrum of I_{3d} for I_2-HPCM-NP (Supplementary Fig. 12) exhibited a binding energy shift about 0.6 eV toward lower binding energy in comparison with that of the pure I_2, corresponding to the increase of charge density[18,19]. This is in aggrement with the theoretical calculation (Supplementary Fig. 13). The nitrogen and phosphorous doping reuslted in the charge relocalization when the iodine molecules were adsorbed on the surface of graphene.

Supplementary Note 4. Detailed discussion on theoretical results.

On the basis of the theoretical results, it can be seen that the pure graphene with homogeneous nonpolar surface basically cannot afford a sufficient binding and confining effect to maintain iodine molecules due to the weak interactions (Supplementary Fig. 13a). In contrast, the heteroatom doping using N or P dopant significantly enhances the interaction between the carbon hosts and the iodine guests. The adsorption of iodine is quite sensitive to dopant elements according to the largely varied bond length of iodine molecule, adsorption energy (E_{ad}), and the differencial charge density. For the nitrogen-doped graphene, the bond length of iodine molecule is stretched from 2.70 to 2.91 Å. The adsorption energy is increased to 0.29 eV from 00.02 eV for the pure graphene while the vertical distance between the adsorption pair decreaes. The charge difference shows that the obvious charge transfer occurs from the substrate to the iodine molecules (Figure 13f). These results suggest the enhanced interactions caused by nitrogen doping in comparison with the pure graphene. In the case of phosphorus doping, the strong interaction between iodine and phosphorus dopant with large bond energy of 5.82 eV leads to the irreversible dissociation of iodine. The charge transfer occurs from iodine to phosphorus (the green and yellow region coating on the P atoms), indicating the formation of P-I ionic bonds. Fortunately, the strong-coupling effect can be achieved via N and P co-doping. As demonstrated in Supplementary Fig. 13d,e, the bond length of iodine is 2.96 and 2.93 Å on the graphene co-doped with isolated N and P, and coupled N and P, respectively. The adsorption energy of 2.39 and 2.89 eV suggests the formation of stable chemisorption. Meanwhile, the charge density indicates that most of the charge accumulates in the surrounding of the I atoms (green region in Figures 13f (IV and V)), suggesting the strong chemical interactions between iodine and the carbon substrate. Therefore, the strong interactions toward iodine would benefit to the nucleation of iodine by lowering the surface tensile against the carbon substrate, leading to the a high loading of iodine on the HPCM-NP electrode. These results prove that the doping of carbon with N and P helps to anchor iodine onto the doped carbon substrate, and thereby effectively suppress the shuttle effect during cycling of the batteries, which is in good agreement with our experimental testing.

The adsorption energy (E_{ad}) of LiI_3 on different carbon substrates, the distance between the adsorption pair, and the varied bond lengths of I-I were calculated. In comparison with the pure graphene, the nitrogen doping could change the electronic structure of adjacent carbon atoms to promote the interaction between LiI_3 and the doped graphene with enhanced adsorption energy (1.02 vs 0.97 eV) and shorter vertical distance (2.21 vs 2.24 Å). The phosphorus doping leads to the irreversible dissociation of LiI_3 molecular with a large adsorption energy of 5.42 eV. The adsorption energy is 2.88 and 3.46 eV on the graphene doped with isolated N and P, and coupled N and P, respectively. The striking adsorption energy with close contact (1.70 and 2.19 Å) exhibits the synergic effect of nitrogen and phosphorous co-doping, which could ensure the suitable interaction of LiI_3 to the N, P-co-doped carbon matrix. These results suggest the nitrogen and phosphorus doping is beneficial to the adsorption and subsequent redox reactions between LiI_3 and I_2 at the interface of the carbon electrode and electrolyte.

Supplementary Note 5. The cycling stability test in a glassy bottle.

The cycling stability of the Li-I_2 battery was tested in a glassy bottle (Supplementary Fig. 18). It can be seen that only slight color change caused by the iodine was observed after 1000 charge/discharge cycles. The capacity retention was around 80.2%, suggesting an acceptable cycling stability. At the same time, the iodine content in the electrolyte was measured to be only 9.5 % after cycling stability test. The low ratio suggests the intercalation of Li or Na ions did not significantly deteriorate the stability of iodine.

Supplementary Note 6. The performance comparison of HPCM-NP electrodes for Li and Na storage.

As demonstrated, the specific capacity of Li-I_2 battery is larger than that of Na-I_2 battery althrough the same I_2-carbon electrode was used on the basis of the similar redox reactions between I_2 and Li/Na. These results indicate the possible influence of the ion intercalation on the specific capacity of the carbon electrode due to the different ionic radius between Li^+ and Na^+. When used as cathodes, the specific capacity of HPCM-NP is only around 56 and 37 mAh g^{-1} for Li^+ and Na^+ storage (Supplementary Fig. 20).

Supplementary Note 7. Electrochemical impedance spectroscapy test and Dynamic contact angle analysis.

The free-standing I_2-HPCM-NP composite with a highly conductive carbon sketch could provide rapid charge transfer pathways. It is evidenced by the smaller charge transfer resistance (R_{ct}, 42 Ω) for the I_2-HPCM-NP electrode in comparison with that of the I_2-AC electrode (R_{ct}, 89 Ω) according to the electrochemical impedance spectroscopy (Supplementary Fig. 22a). Furthermore, the highly conductive carbon fiber as the inner core would be beneficial to providing rapid electron transfer pathway. The contact angle tests with a Dynamic Contact Angle Analyzer (Supplementary Fig. 22b) clearly revealed the good wettability of the I_2-HPCM-NP for the electrolyte. Such good wettability could lead to the facile penetration and fast diffusion of electrolytes into the porous electrode, which is of importance for achieving a good battery performance. As demonstrated in Supplementary Fig. 22c (down), the intimate interaction between the substrate and carbon particles would be possible. However, it is hard to form an efficient charge transfer route among the carbon particles, resulting in a relatively large charge-transfer resistance. In contrast, the 3D hierarchical interconnected porous framework not only allows the electrolyte easy accessibility to the inner surface but also ensures effective electronic transport pathways along the conductive carbon sketch. Therefore, 3D hierarchically porous HPCM-NP electrode with a larger surface area is proved to be useful in minimizing electron and ion transport resistance due to the bicontinuous porous structure, which is also in good agreement with the previously reported results.

Supplementary Note 8. The stability test of iodine adsorbed HPCM-NP electrode during the cycling test according to the UV-Vis spectrocopy.

As known in Supplementary Fig. 24a, the optical absorption spectra exhibit a characteristic absorption peak of I_2 at 220 nm in ethanol solution[20]. The absorbance at 220 nm was recorded for each sample and plotted versus the iodine content of the solution. According to the Lambert-Beer law ($A = \varepsilon \times b \times c$), a linear relationship between the concentration of iodine (c) and the absorbance of the typical peak (A) was obtained, which was used to determine the content of iodine in the electrolyte after cycling (Supplementary Fig. 24b). Typically, each cell was stopped at full charged state after cycling test to ensure the iodine species in I_2 state for spectrum characterization. The stainless steel coin cell and separator were washed with ethanol. The obtained solution was analyzed to examine the content of iodine. The content of iodine in the electrolyte after 2000 cycles was given in the inseted note in Supplementary Fig. 24b. The calculated mass loss rate is about 4.0%, confirming the slow dissolution of the iodine.

Supplementary Note 9. The stability test of electrolyte/electrode according to cyclic voltammetry.

To examine the stability of electrolyte/electrode, the cyclic voltammetry curve of HPCM-NP electrode (Supplementary Fig. 25a) exhibited the nearly perfect rectangle shape without any redox peak in a voltage range of 2.0-4.0 V (vs. Li^+/Li) at a low scan rate of 0.1 mV s^{-1}, suggesting a good stability of the electrode material and electrolyte without side reaction. The typical peaks for I_2-HPCM-NP are ascribed to the reversible redox of iodine (Supplementary Fig. 25b). Therefore, the battery with I_2-HPCM-NP electrode performed well in the potential range of 2.0-3.6 V.

Supplementary Note 10. Surface morphologies of Li-metal electrode before and after the cycling test.

The surface morphologies of Li-metal electrode were characterized by using scanning electron microscopy. As shown in Supplementary Fig. 26a, the surface of the pristine Li-metal is uniform in general except some scratches. However, after 2000 cycles, the surface became rough with the Li dendritic structures. The growth of needle-like sharp lithium dendrites could pierce through the separator to cause short circuit and even explosion. Along the extensive research and development on advanced approaches to address this issue for safe applications of Li-metal batteries, it is also highly desirable to construct advanced battery systems without the unsafe metallic Li or Na electrode, as demonstrated in the present study.

Supplementary Note 11. Detailed discussion on anodic performance of HPCM-NP.

In the first CV cycle, obvious cathodic peaks were observed at 0.6 V (vs. Li^+/Li, Supplementary Fig. 29a) and 0.5 V (vs. Na^+/Na, Supplementary Fig. 29b). These peaks were analogy attributed to the decomposition of the electrolyte and/or the formation of solid electrolyte interphase (SEI) layer[21-23]. The initial discharge/charge capacities of 644/255 mAh g^{-1} for NIB was smaller than that for LIB due to the larger ionic radius of Na^+ ion. reproduces For the discharge/charge curves of the Li//HPCM-NP and Na//HPCM-NP half-cell at 100 mA g^{-1} in the voltage range of 0.01-3 V (Supplementary Fig. 29c-d) show the apparent plateaus in the first discharge process for Li and Na cells. Typically, the Li//HPCM-NP and Na//HPCM-NP exhibited the initial discharge/charge capacities of 922/388, 644/255 mAh g^{-1} with an initial Coulombic efficiency of 42.1, 39.6%, respectively. After 500 cycles, 68.8, 65.1% of the reversible discharge capacity for LIB and NIB were preserved. Notably, the HPCM-NP also exhibited excellent cycling stability and good high-rate performance. Even at a high current density of 2000 mA g^{-1}, a capacity retention of 77.3/62.4% of the nominal capacity for Li^+/Na^+, respectively, were achieved (Supplementary Fig. 29e-f). When returned to the initial current density of 100 mA g^{-1}, the capacities of Li-ion and Na-ion half-cell were restored to 387 mAh g^{-1} (99.6%, capacity recuperation) and 158 mAh g^{-1} (96.6%, capacity recuperation), suggesting the good high-rate performance. Besides, the long-term stability test also exhibited good capacity retention after 2000 cycles (Supplementary Fig. 29g-h).

Supplementary Note 12. The full battery performance using a HPCM-NP electrode.

With the favorable $LiMn_2O_4$ or $Na_3V_2(PO_4)_3/C$ cathode (Supplementary Fig. 30), respectively, Li/Na-ion full cells have been fabricated by coupling with a HPCM-NP electrode. The Li-ion battery exhibited a specific capacity of around 83 mAh g^{-1} whereas the specific capacity for the Na-ion battery was only 70 mAh g^{-1} on the basis of the total mass of anode and cathode. The Li-ion battery also exhibited a better high rate performance and an excellent cycling stability (~79% after 500 cycles, Supplementary Fig. 31) in comparison with Na-ion battery. These results demonstrate the good intercalative properties of HPCM-NP that is related to the ion radius (e.g., Li^+ and Na^+) and intercalative properties of cathodes. Thus, it is high promising to optimize the ion intercalative capability of HPCM-NP electrode in a full cell.

Supplementary References

1 Liu, F. C., Liu, W. M., Zhan, M. H., Fu, Z. W. & Li, H. An all solid-state rechargeable lithium-iodine thin film battery using LiI(3-hydroxypropionitrile)$_2$ as an I^- ion electrolyte. *Energy Environ. Sci.* **4**, 1261-1264 (2011).

2 Su, Z., Tong, C.-J., He, D.-Q., Lai, C., Liu, L.-M., Wang, C. & Xi, K. Ultra-small B_2O_3 nanocrystals grown in situ on highly porous carbon microtubes for lithium-iodine and lithium-sulfur batteries. *J. Mater. Chem. A* **4**, 8541-8547 (2016).

3 Wang, Y. L., Sun, Q. L., Zhao, Q. Q., Cao, J. S. & Ye, S. H. Rechargeable lithium/iodine battery with superior high-rate capability by using iodine-carbon composite as cathode. *Energy Environ. Sci.* **4**, 3947-3950 (2011).

4 Zhao, Q., Lu, Y., Zhu, Z., Tao, Z. & Chen., J. Rechargeable lithium-iodine batteries with odine/nanoporous carbon cathode. *Nano Lett.* **15**, 5982-5987 (2015).

5 Etacheri, V., Wang, C., O'Connell, M. J., Chan, C. K. & Pol, V. G. Porous carbon sphere anodes for enhanced lithium-ion storage. *J. Mater. Chem. A* **3**, 9861-9868 (2015).

6 Penki, T. R., Shanmughasundaram, D., Kishore, B. & Munichandraiah, N. High rate capability of coconut kernel derived carbon as an anode material for lithium-ion batteries. *Adv. Mater. Lett.* **5**, 184-190 (2014).

7 Wang, L., Schütz, C., Salazar-Alvarez, G. & Titirici, M.-M. Carbon aerogels from bacterial nanocellulose as anodes for lithium ion batteries. *RSC Adv.* **4**, 17549-17554 (2014).

8 Selvamani, V., Ravikumar, R., Suryanarayanan, V., Velayutham, D. & Gopukumar, S. Garlic peel derived high capacity hierarchical N-doped porous carbon anode for sodium/lithium ion cell. *Electrochim. Acta* **190**, 61-67 (2014).

9 Zhang, W., Yin, J., Lin, Z. Lin, H., Lu, H., Wang, Y. & Huang, W. Facile preparation of 3D hierarchical porous carbon from lignin for the anode material in lithium ion battery with high rate performance. *Electrochim. Acta* **176**, 1136-1142 (2015).

10 Komaba, S., Murata, W., Ishikawa, T., Yabuuchi, N., Ozeki, T., Nakayama, T., Ogata, A., Gotoh, K. & Fujiwara, K. Electrochemical Na insertion and solid electrolyte interphase for hard-carbon electrodes and application to Na-ion Batteries. *Adv. Funct. Mater* **21**, 3859-3867 (2011).

11 Cao, Y. L., Xiao, L. F., Sushko, M. L., Wang, W., Schwenzer, B., Xiao, J., Nie, Z. M., Saraf, L. V., Yang, Z. G. & Liu, J. Sodium ion insertion in hollow carbon nanowires for battery applications. *Nano Lett.* **12**, 3783-3787 (2012).

12 Wang, H. G., Wu, Z., Meng, F. L., Ma, D. L., Huang, X. L., Wang, L. M. & Zhang, X. B. Nitrogen-doped porous carbon nanosheets as low-cost, high-performance anode material for sodium-ion batteries. ChemSusChem **6**, 56-60 (2013).

13 Fu, L., Tang, K., Song, K., Van Aken, P. A., Yu, Y. & Maier, J. Nitrogen doped porous carbon fibres as anode materials for sodium ion batteries with excellent rate performance. *Nanoscale* **6**, 1384-1389 (2014).

14 Chen, T., Liu, Y., Pan, L., Lu, T., Yao, Y., Sun, Z., Chua, D. H. C. & Chen, Q. Electrospun carbon nanofibers as anode materials for sodium ion batteries with excellent cycle performance. *J. Mater. Chem. A* **2**, 4117-4121 (2014).

15 Wang, Z., Qie, L., Yuan, L., Zhang, W., Hu, X. & Huang, Y. Functionalized N-doped interconnected carbon nanofibers as an anode material for sodium-ion storage with excellent performance. *Carbon* **55**, 328-334 (2013).

16 Zhang, J., Zhao, Z., Xia, Z. & Dai, L. A metal-free bifunctional electrocatalyst for oxygen reduction and oxygen evolution reactions. *Nat. Nanotechnol.* **10**, 444-452 (2015).

17 Pan, L. J., Yu, G., Zhai, D., Lee, H. R., Zhao, W., Liu, N., Wang, H., Tee, B. C. K., Shi, Y., Cui, Y. & Bao, Z. N. Hierarchical nanostructured conducting polymer hydrogel with high electrochemical activity. *Proc. Natl Acad. Sci. USA* **109**, 9287-9292 (2012).

18 Tian, H., Gao, T., Li, X., Wang, X., Luo, C., Fan, X., Yang, C., Suo, L., Ma, Z., Han, W. & Wang, C. High power rechargeable magnesium/iodine battery chemistry. *Nat. Commun.* **8**, 14083 (2017).

19 Zhou, G., Tian, H., Jin, Y., Tao, X., Liu, B., Zhang, R., Seh, Z. W., Zhuo, D., Liu, Y., Sun, J., Zhao, J., Zu, C., Wu, D. S., Zhang, Q. & Cui, Y. Catalytic Oxidation of Li_2S on the Surface of Metal Sulfides for Li-S Batteries. *Proc. Natl. Acad. Sci. U. S. A.* **114**, 840-845 (2017).

20 Custer, J. J. & Natelson, S. Spectrophotometric determination of microquantities of iodine. *Anal. Chem.* **21**, 1005-1009 (1949).

21 Zhang, C., Mahmood, N., Yin, H., Liu, F. & Hou, Y. Synthesis of phosphorus-doped graphene and its multifunctional applications for oxygen reduction reaction and lithium ion batteries. *Adv. Mater.* **25**, 4932-4937 (2013).

22 Xu, J., Lin, Y., Connell, J. W. & Dai, L. Nitrogen-doped holey graphene as an anode for lithium-ion batteries with high volumetric energy density and long cycle life. *Small* **11**, 6179-6185 (2015).

23 Lu, K., Xu, J., Zhang, J., Song, B. & Ma, H. General preparation of three-dimensional porous metal oxide foams coated with nitrogen-doped carbon for enhanced lithium storage. *ACS Appl. Mater. Interfaces* **8**, 17402-17408 (2016).
